# Supplementary material for: Autocatalytic and oscillatory reaction networks that form guanidines and products of their cyclization
Source: Nat Commun. 2021 May 20;12:2994. doi: 10.1038/s41467-021-23206-9 (PMC8138026; doi:10.1038/s41467-021-23206-9)
Supplement: Supplementary file 1 — Supplementary Information [file 41467_2021_23206_MOESM1_ESM.pdf]

Supplementary Information for

Autocatalytic and Oscillatory Reaction Networks that Form Guanidines and Products of their Cyclization

Alexander I. Novichkov, Anton I. Hanopolskyi, Xiaoming Miao, Linda J. W. Shimon, Yael Diskin-Posner, and Sergey N. Semenov*

*Correspondence to: sergey.semenov@weizmann.ac.il

Table of Content

1. Materials and Methods 3

2. Synthesis 4

3. Single crystal X-ray diffraction analysis 12

4. Batch kinetic experiments 14

4.1. General protocol for ^{1}H NMR kinetics experiments 14

4.2. Fitting of the rate constants 14

4.3. Batch experiments showing a pulse 19

5. Supplementary discussion of the one-pot cyclization reactions 21

5.1. Formation of bicyclic products via tandem cyclization 21

5.2. Oxidation of the dihydropyrimidine derivative 24 to pyrimidine derivative 25 34

5.3. One-pot formation of the aminopyrimidine derivative 26 35

6. Flow experiments 36

6.1. Flow setup 36

6.2. Standard protocol for oscillations in flow 39

6.3. Protocol for studies of responses of oscillators to chemical stimuli 40

6.4. Data processing for the oscillatory experiments 41

6.5. Plots of oscillations for all experiments 42

7. Modeling 46

7.1. Model of the autocatalytic reactions 46

7.2. Complete model of the oscillator 50

7.3. Three-variable model and linear stability analysis of the oscillator 53

8. NMR spectra 55

9. Supplementary references 60

1. Materials and Methods

Thiourea, sodium 2-bromoethanesulfonate, methyl chloroformate, potassium thiocyanate, piperidine, bromoethane, 3-bromopropane-1amine hydrobromide, ethanethiol, sodium thiosulfate, iodine, potassium phtalimide, 1,4-dibromobutane, potassium ethyl xanthogenate, hydrazine hydrate, (2-Bromoethyl)trimethylammonium bromide, monopotassium phosphate (KH_2PO_4), dipotassium phosphate (K_2HPO_4), potassium carbonate (K_2CO_3), and potassium hydroxide (KOH) were purchased from Sigma-Aldrich, Acros Organics, Alfa Aesar, and Merck. Solvents – dichloromethane (DCM), methanol, ethanol, n-hexane, ethyl acetate, acetone, and anhydrous DMF were purchased from Sigma-Aldrich and Acros Organics. D_2O was purchased form Tzamal d-chem; all other NMR solvents were purchased from Cambridge Isotope Laboratories. All chemicals, including solvents, were used without further purification. LC-MS grade water was used in all kinetic experiments. 2-ethylisothiouronium bromide (**5**) was prepared according to the literature procedure.[1] *General warning! Thios have strong unpleasant smell. All work has to be conducted in a well-ventilated hood.*

NMR spectra were measured on a Bruker AVANCE III-300 spectrometer at 300 MHz for ^{1}H and 73.7 MHz for ^{13}C{1H}, on a Bruker AVANCE III-400 spectrometer at 400 MHz for ^{1}H and 100.6 MHz for ^{13}C{1H}, and on a Bruker AVANCE III HD-500 spectrometer at 500 MHz for ^{1}H and 125.8 MHz for ^{13}C{1H}. Chemical shifts for ^{1}H and ^{13}C are given in ppm relative to TMS. ^{1}H and ^{13}C spectra were calibrated using a residual solvent peak as an internal reference DMSO-d^{6} (^{1}H NMR: δ = 2.50 ppm, ^{13}C NMR: δ = 39.52 ppm), D_2O (^{1}H NMR: δ = 4.79 ppm). Data for the ^{1}H NMR spectra were reported as follows: chemical shift (ppm), peak shape (s = singlet, d = doublet, t = triplet, q = quartet, p = pentet, m = multiplet, br = broad, dd = doublet of doublets), coupling constant (Hz), and integration.

The absorbance in the flow experiments was constantly monitored on a Cary 60 UV-VIS spectrometer, manufactured by Agilent Technologies. The flow cells used in these experiments was either a commercial flow cell with a light path of 0.2 mm, purchased from Hellma Analytics or a hand-made cell (see the section describing the flow set-up). The reactants in the flow experiments were supplied by NEMESYS Low Pressure Syringe pumps (Gear: 14:1, Type: NEM-B101-02E), produced by CETONI GmbH.

The Chromatographic separation and mass analysis were performed on a Waters Acquity liquid chromatography system equipped with a PDA Detector (210 and 700 nm) and a Waters QDa mass detector with an electrospray ionization (ESI) and a mass range of 85–1250 m/z.

2. Synthesis

2-((amino(iminio)methyl)thio)ethane-1-sulfonate (**3**)

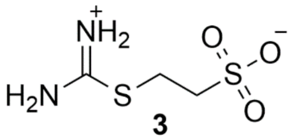

3

Compound (**3**) was synthesized using a modified literature procedure.[2] Briefly, 2 g (9.5 mmol) of sodium 2-bromoethanesulfonate was dissolved in 25 ml of DMF, together with 870 mg of thiourea (11.5 mmol, 20% excess). The reaction mixture was stirred at 90 °C for 10 hours. Solvent was removed under vacuum and the solid part was recrystallized twice from a water-methanol mixture (1:1). Finally, 940 mg of white crystalline product was obtained (yield 54%). ^{1}H NMR (300 MHz, DMSO-d^6) δ = 9.14-8.85 (br, 4H), 3.34 (t, J = 7.3 Hz, 2H, overlaps with H_2O admixture), 2.80 (t, J = 7.3 Hz, 3H). ^{13}C NMR (73.7 MHz, DMSO-d^6) δ = 171.04, 50.55, 27.24.

*S-phenylisothiouronium bromide (**4**)*

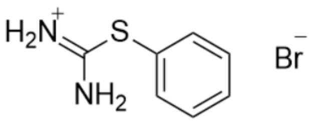

(4)

S-phenylisothiouronium bromide was synthesized by a reaction between thiophenol and cyanamide in diethyl ether according to the literature procedure.[3] The product was converted to hydrobromide before recrystallization. ^{1}H NMR (300 MHz, D_2O) δ = 7.67-7.50 (m, 5H). ^{13}C NMR (73.7 MHz, D_2O) 140.47, 136.43, 132.37, 130.7.

Piperidine-1-carbothioamide

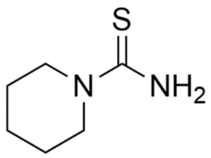

Piperidine-1-carbothioamide was synthesized using a modified literature procedure.[4] A 250 ml round-bottom flask equipped with a condenser was filled with 100 ml of acetone, 7.73 ml of methyl chloroformate (0.1 mole), and 9.7 g (0.1 mole) of potassium thiocyanate. The mixture was refluxed for 2 hours and after having been cooled, 11.8 ml of piperidine (0.12 mole) were added dropwise. The reaction mixture was stirred for another hour at room temperature and then treated with 200 ml of 6M HCl, evaporated, and the resulting light yellow oil was recrystallized from isoprapanol. Finally, 8.35 g of crystalline product were obtained (yield

58%) ^{1}H NMR (300 MHz, $CDCl_3$) δ = 5.58 (br , 2H), 3.75 (br, 4H), 1.64 (br, 6H). ^{13}C NMR (73.7 MHz, $CDCl_3$) δ = 180.81, 49.38 (br), 25.44, 23.84.

Ethyl piperidine-1-carbimidothioate hydrobromide (**7**)

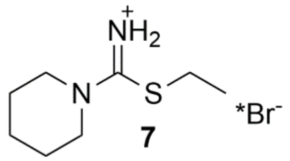

7

Thiouronium salt (**7**) was synthesized by a reaction between piperidine-1-carbothioamide and bromoethane. The procedure was the same as for the synthesis of **5**.[1] Briefly, 5 g of piperidine-1-carbothioamide (35 mmole) was dissolved in 50 ml of methanol and 3ml of bromoethane (42 mmole, 20% excess) was added. The reaction mixture was refluxed with stirring for 6h and after having been cooled, the solvent was removed under vacuum. Solid residue was recrystallized from H_2O:i-PrOH (1:3). Finally, 6.7 g of crystalline compound were obtained (yield 76%) ^{1}H NMR (300 MHz, DMSO-d^6) δ = 9.07 (br , 2H), 3.73 (br, 4H), 3.31 (q, J = 7.2 Hz, 2H), 1.62 (br, 6H), 1.27 (t, J = 7.2 Hz, 3H). ^{13}C NMR (73.7 MHz, DMSO-d^6) δ = 165.70, 27.03, 25.76 (br), 23.34, 14.31.

Disulfide of 3-aminopropane-1-thiol

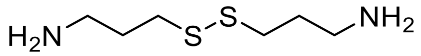

A solution of 3-bromopropane-1amine hydrobromide (3.00 g, 13.7 mmole) and $Na_2S_2O_3$ (3.74 g, 15.1 mmole) were dissolved in a mixture of MeOH/H_2O (1/1, 20 mL). This solution was heated to 90°C and stirred for 3 hours. Iodine (1.91 g, 7.54 mmole) in MeOH was slowly added via a dropping funnel for over 1 hour. The mixture was left stirring for 1 additional hour and then the mixture was concentrated under reduced pressure. The residue was dissolved in 6N aqueous NaOH (10 mL) and then 50 mL of DCM was added. The layers were separated and one more round of extraction was conducted. The organic layers were dried over Na_2SO_4 and evaporated. The residue was treated with HCl and recrystallized to afford the desired hydrochloride of the disulfide of 3-mercaptopropane-1amine. Yield: 1.27 g (5 mmole, 37%). %). ^{1}H NMR (300 MHz, D_2O) δ = 3.01 (t, J = 6.6 Hz, 4H), 2.7 (t, J = 7.3 Hz, 4H), 1.99 (m, 4H). ^{13}C NMR (73.7 MHz, D_2O) δ = 37.99, 33.59, 25.88.

3-aminopropane-1-thiol (**9**)

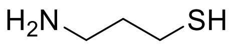

9

Disulfide of 3-aminopropane-1-thiol from the previous experiment (500 mg, 2.7 mmole) in the form of a free base was dissolved in 15 ml of Et_2O and mixed with 1 ml of EtSH (13 mmole). The vial with the reaction

mixture was kept in a refrigerator for 24 h. Next, 3-aminopropane-1-thiol formed as flat hexagonal crystals on the walls of the vial. Next, the crystals were washed with diethyl ether and dried under *vacuo*. Yield: ~300 mg (60%). ^{1}H NMR (300 MHz, DMSO-d^6) δ = 2.60 (t, J = 6.6 Hz, 2H), 2.51 (t, J = 7.0 Hz, 2H), 1.59 (p, J = 6.8 Hz, 2H). ^{13}C NMR (73.7 MHz, DMSO-d^6) δ = 40.51, 37.73, 21.82.

S-ethyl carbamohydrazonothioate **(6)**

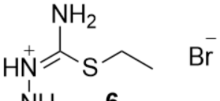

6

A solution of thiosemicarbazide (5.0 g 55.5 mmole) was dissolved in 50 ml of methanol together with 5ml (65 mmole) of bromoethane. Next, the mixture was refluxed for 8 hours. After removal of the solvent, the dry part was recrystallized from the ethanol/water mixture (1:1). Yield: 6.2 g (66%). ^{1}H NMR (300 MHz, DMSO-d^6) δ = 10.67 (br, 1H), 9.19 (br, 2H), 5.24 (br, 1H), 3.18 (br, 2H), 1.23 (t, J = 7.3 Hz, 3H). ^{13}C NMR (73.7 MHz, DMSO-d^6) δ = 166.32 (br), 25.39, 14.90.

N-(4-bromobutyl)phtalimide

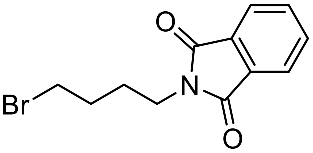

This compound was synthesized using a modified literature procedure.[5] Potassium phtalimide (1.86 g, 10 mmole) was added to the solution of 1,4-dibromobutane (30 mmole, 3.9 mL) in DMF (10 mL) at room temperature. The reaction mixture was stirred at 80°C for 24 hours. Then, it was filtered and washed with 2x20 mL of DMF. The filtrate was concentrated in *vacuo* and poured into an ice-water bath. The white solid, which precipitated from the water, was recrystallized from methanol to afford the desired product. Yield: 2.25 g, 80%. ^{1}H NMR (300 MHz, DMSO-d^6): δ = 7.83 (m, 4H), 3.60 ppm (t, J = 6.5 Hz, 2H), 3.55 (t, J = 6.5 Hz, 2H), 1.79 (m, 2H), 1.71 (m, 2H). The spectra data were in agreement with the values reported in the literature. [5]

S-(4-(1,3-dioxoisoindolin-2-yl)butyl) O-ethyl carbonodithioate

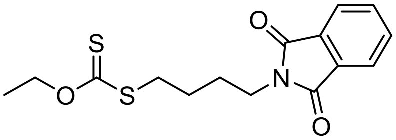

This compound was synthesized using a modified literature procedure.[6] N-(4-bromobutyl)phtalimide (0.47 g, 1.7 mmole), obtained in the previous step, was dissolved in 1 mL of acetone and a suspension of potassium ethyl xanthogenate (0.533 g, 3.4 mmole) in 1 mL of acetone was added in the course of 15 minutes. The reaction mixture was stirred overnight and then was quenched with water (10 mL) and extracted with

DCM. The organic phase was dried with Na_2SO_4 and evaporated in *vacuo*. The product was purified by gradient column chromatography (Hex/EtOAc, 0-25%). Yield: 1.5 g (43%). ^{1}H NMR (300 MHz, $CDCl_3$): δ = 7.86 (m, 2H), 7.75 (m, 2H), 4.65 (q, J = 7.1 Hz, 2H), 3.73 (t, J = 6.7 Hz, 2H), 3.18 (t, J = 6.7 Hz, 2H), 1.80 ppm (m, 4H), 1.42 ppm (t, J = 7.1 Hz, 3H).

4-aminobutane-1-thiol (**10**)

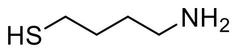

10

Compound **10** was synthesized using a modified literature procedure.[6] Xanthogenate derivative, obtained in the previous stage (1 g, 3.1 mmole), was refluxed with $N_2H_4 \cdot H_2O$ (5 mL) in 20 mL of degassed EtOH in an argon atmosphere. Refluxing continued for 3h before the temperature was lowered to room temperature and the reaction mixture was left under stirring overnight. The white precipitate, which formed overnight, was filtered and washed with EtOH. The filtrate was concentrated. A slightly yellow solid that formed during the evaporation of EtOH was dissolved in 5 mL of H_2O, and the water solution was extracted with DCM (3x10 mL). Evaporation of DCM afforded a yellow liquid. This liquid was dissolved in an excess of diethyl ether, and the desired product was precipitated with 4M HCl in dioxane (1 mL). It was then washed with acetonitrile and recrystallized from ethanol. Yield: 210 mg (48%) ^{1}H NMR (300 MHz, D_2O): δ = 2.91 (t, J = 6.5 Hz, 2H), 2.66 (t, J = 6.5 Hz, 2H), 1.67 (m, 4H). ^{13}C NMR (73.7 MHz, D_2O): δ = 39.1, 36.9, 25.5, 25.2.

2-(2-(trimethylammonio)ethyl)isothiouronium dibromide (**8**)

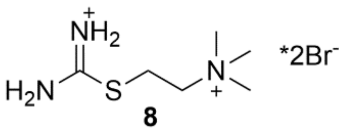

8

Substance **8** was obtained according to the modified literature procedure.[7] The reaction was carried out in a vial equipped with a magnetic stirring bar. The mixture of (2-bromoethyl)trimethylammonium bromide (500 mg, 2.02 mmol) and thiourea (161 mg, 2.12 mmol) (1: 1.05) in 3 ml of methanol was stirred at 65 ° C for 48 hours. After solvent evaporation, the solid part was recrystallized twice from methanol (20 ml of hot methanol for each recrystallization). Yield 550 mg (84%) ^{1}H NMR (500 MHz, D_2O) δ = 3.72 – 3.69 (m, 2H), 3.63 – 3.60 (m, 2H), 3.19 (s, 9H). ^{13}C NMR (125.8 MHz, D_2O) δ = 169.03, 63.69, 53.35, 24.12.

*2-amino-4,5-dihydrothiazol-3-ium-4-carboxylate (**16**)*

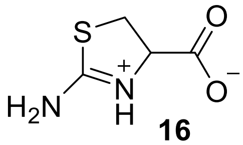

16

Next, 100 mg of (**4**) (0.429 mmole) with 52 mg of cysteine (0.429 mmole) were dissolved in 0.5 ml of phosphate buffer solution in D_2O (1 M, pH 8). We monitored the progress of the reaction using NMR, and after 2 days, the reaction was complete. Insoluble in water, thiophenol was separated and the remaining solution was evaporated. The dry residue was extracted with ethyl acetate (3 times, 0.5ml). The ethyl acetate extract was concentrated. Compound **16** was obtained as crystals suitable for single-crystal X-ray analysis by slow mixing of ethyl acetate extract with diethyl ether (2ml) in NMR tube. ^{1}H NMR (400 MHz, D_2O) δ = 4.25 (dd, J = 6.2, 4.4 Hz, 1H), 3.58 (dd, J = 14.1, 4.4 Hz, 1H), 3.58 (dd, J = 15.0, 6.2 Hz, 1H). MS: $[M+H]^+$ calcd. for $C_4H_6N_2O_2S$: 147.0; found 147.1.

*1-(2-mercaptoethyl)guanidine hydrobromide (**11**).*

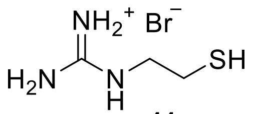

11

1.26 g (6.77 mmole) of 2-ethylisothiouronium bromide, 0.76 g (6.77 mmole) of cysteamine hydrochloride, and 2.35 g (13.53 mmole) of K_2HPO_4 were charged into 50 mL flask. The system was evacuated and backfilled with argon three times to remove oxygen. The mixture was dissolved in 7 mL of degassed H_2O. After 10 minutes of stirring the solution became transparent. The reaction was monitored by ^{1}H NMR. After 6 hours the conversion reached 90% and the reaction was left overnight. After the signals of cysteamine disappeared, HCl was added to reach the pH=3-4. The solution was concentrated to 5-10% of initial volume. Then ethanol (30 mL) was added to precipitate inorganic salts. After filtration the solid was washed three times with ethanol (10 mL). Combined washings were concentrated and the product obtained as a transparent slurry, which was used for the next steps without further purification. Yield: 0.96 g (92%). ^{1}H NMR (500 MHz, D_2O): δ 3.32 (t, J=6.65 Hz, 2H), 2,65 (t, J=6.65 Hz, 2H). ^{13}C NMR (75 MHz, D_2O): δ 43.93, 22.91, 156.8. MS: $[M+H]^+$ calcd. for $C_3H_{10}N_3S$: 120.1; found 120.2.

*1,1'-(disulfanediylbis(ethane-2,1-diyl))diguanidine dihydroiodide (**18**).*

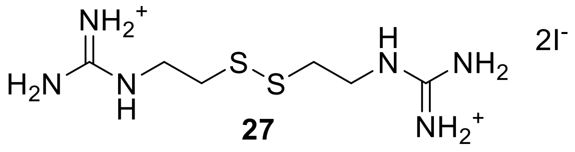

27

0.5 g (3.2 mmole) of 2-mercaptoethyl-guanidine was mixed with 5 mL of MeOH. The solution of I_2 was prepared by dissolving 0.816 g (3.2 mmole) of I_2 in MeOH (10 mL). Then the I_2 solution had been added

dropwise to the solution of guanidine on stirring. The addition stopped when the yellow color of I_2 in the reaction mixture remained for 5 minutes. The mixture was immediately concentrated in *vacuo*. Obtained solid was washed three times with benzene and dried. Slightly yellow solid was recrystallized twice from ethanol to afford the desired product. Yield: 320 mg (65%). ^{1}H NMR (500 MHz, D_2O): δ 3.49 (t, J=6.37 Hz, 2H), 2.86 (t, J=6.37 Hz, 2H). ^{13}C NMR (125 MHz, D_2O): 39.72, 35.95, 156.8. MS: calcd. for $[M+H]^+$ $C_6H_{17}N_6S_2$: 237.1; found 237.3, calcd. for $[M+2H]^{2+}$ $C_6H_{18}N_6S_2^{2+}$: 119.05; found: 119.2.

*2,2'-((disulfanediylbis(ethane-2,1-diyl))bis(azanediyl))bis(6-methylpyrimidin-4(3H)-one) (**19**).*

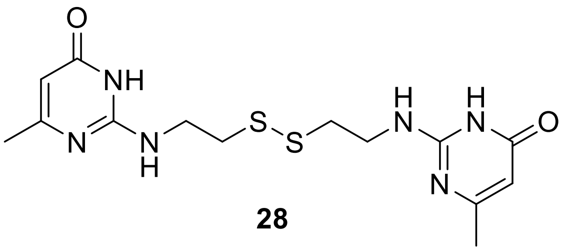

28

200 mg (0.64 mmole) of disulfide **18** and 196 mg of K_2CO_3 (2.2 eq, 1.42 mmole) were stirred in EtOH for 10 minutes. Then ethyl acetoacetate (180 mg, 178 µL, 1.1 eq) was added. The mixture was left to reflux overnight. Then EtOH was evaporated and the inorganic salts were washed out by MeOH. The methanol solution was concentrated to give white solid. Half of this solid was separated using the column chromatography (Silica-60, mesh 0.40-0.63, eluent DCM/MeOH, 15:1) to give compound **19**. Yield: 10 mg (13% considering that only half of the reaction mixture was purified). Crystals suitable for X-ray analysis were grown by vapor diffusion of THF into methanol solution of **19**. ^{1}H NMR (500 MHz, CD_3OD): δ = 5.63 (s, 2H), 3.72 (t, J=6.78 Hz, 4H), 2.98 (t, J=6.78 Hz, 4H), 2.17 (6H, s), ^{13}C NMR (125 MHz, CD_3OD): 100.65 (br), 39.38, 37.07, 29.35 (br). HRMS: calcd. for $[M+H]^+$ $C_{14}H_{21}N_6O_2S_2^+$ 369.1161; found 369.1156; calcd. for $[M+Na]^+$ $C_{14}H_{20}N_6O_2S_2Na^+$ 391.09814; found 391.0976.

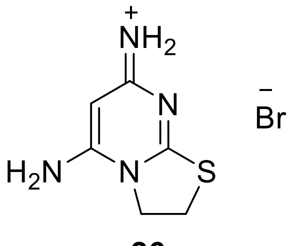

26

Preparative synthesis of *7-imino-2,3-dihydro-7H-thiazolo[3,2-a]pyrimidin-5-amine* **(26)**. *Thiazolidin-2-imine* was prepared according to the previously known procedure.[8] Thiazolidin-2-imine hydrobromide (0.5 g, 2.73 mmole) was mixed with 0.54 g (3 eq, 8.5 mmole) of malonitrile and dissolved in 5 mL of ethanol and 2 mL of pyridine. The mixture was refluxed for 6 hours. Then the solvents were concentrated, and 48% HBr was added. The product precipitated and was recrystallized from EtOH/H_2O. Yield: 190 mg (0.76 mmole, 28%). **^{1}H NMR**

(300 MHz, DMSO) δ = 7.95 (s, 2H), 7.81-7.4 (br, 2H), 5.49 (s, 1H), 4.39 (t, J=7.70 Hz, 2H), 3.63 (t, 7.70 Hz, 2H). **^{13}C NMR** (75.4 MHz, D_2O) δ = 166.87, 163.1037, 154.67, 77.52, 50.71, 26.295. **HRMS:** calcd. for $[M+H]^+$ $C_6H_9N_4S^+$ 169.0548; found 169.0547;

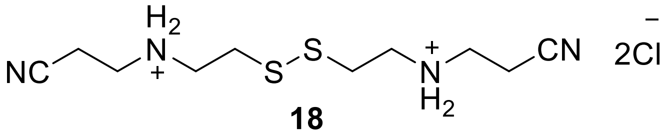

18

Synthesis of *3,3'-((disulfanediylbis(ethane-2,1-diyl))bis(azanediyl))dipropanenitrile* **18**. 2 g of cystamine disulfide dihydrochloride (0.0088 mmole) were dissolved in 20 mL of 10% NaOH and the deprotonated amine was extracted with DCM (3x20 mL), resulting in the slightly yellow oil. This oil was dissolved in DMF (5 mL), then triethylamine 250 µL (20% mol) and acrylonitrile (1.15 mL, 2 eq, 17.6 mmole) were added. The reaction mixture was heated to 70°C and kept at this temperature overnight. Then the obtained mixture was concentrated and separated using gradient flash chromatography (DCM, 0.1% NEt_3/MeOH 0-8%). Obtained transparent oil was dissolved in EtOH and the product was precipitated with HCl as dihydrochloride. Yield: 1.4 g (4.22 mmole, 48%). **^{1}H NMR** (300 MHz, D_2O) δ = 3.42 (t, J=6.80 Hz , 2H), 3.39 (t, J=6.80 Hz, 2H), 2.97 (t, J=6.75 Hz, 2H), 2.93 (t, J=6.75 Hz, 2H). **^{13}C NMR** (75.4 MHz, D_2O) δ = 117.34, 45.94, 42.78, 31.71, 14.72. **HRMS:** ; calcd. for $[M+H]^+$ $C_{10}H_{19}N_4S_2^+$ 259.1051; found 259.1047; calcd. for $[M+Na]^+$ $C_{10}H_{18}N_4NaS_2^+$ 281.0871; found 281.0867.

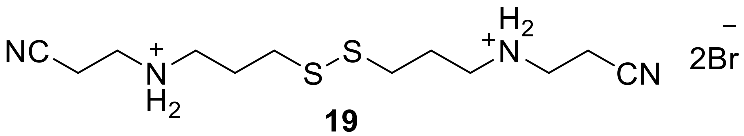

19

Synthesis of *3,3'-((disulfanediylbis(propane-3,1-diyl))bis(azanediyl))dipropanenitrile* **19**.

1.2 g of bis(propan-1-amine)-disulfide (6.67 mmole) was dissolved in DMF (4 mL). Then NEt_3 (190 µL, 20 mol%) and acrylonitrile (880 µL, 2 eq) were added to the solution. The mixture was heated to 70°C and kept at this temperature overnight. After evaporation of solvent and volatile reagents the mixture was separated with gradient flash chromatography (DCM, 0.1% NEt_3/MeOH 0-8%). Obtained yellow oil was concentrated and dissolved in H_2O (10 mL). 2 mL of 48% HBr was added and the solution was concentrated until viscous liquid was obtained. The product was precipitated with EtOH and white solid was collected via filtration, washed with EtOH and dried. Yield 1.2 g (2.67 mmole, 40%). **^{1}H NMR** (300 MHz, D_2O) δ = 3.34 (t, J=6.78 Hz , 2H), 3.14 (dd, J=6.78 Hz, J=15.1 Hz, 2H), 2.90 (t, J=6.72 Hz, 2H), 2.71 (t, J=6.86 Hz, 2H), 2.04 (p, J=7.2 Hz, 2H). **^{13}C**

NMR (75.4 MHz, D_2O) δ = 117.98, 46.304, 42.71, 33.85, 24.56, 14.79. **HRMS:** cd. for $[M+H]^+$ $C_{12}H_{23}N_4S_2^+$ 287.1364; found 287.1373; calcd. for $[M+Na]^+$ $C_{12}H_{22}N_4NaS_2^+$ 309.1184; found 309.1180.

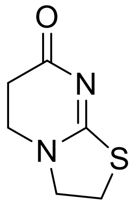

24

Preparative synthesis of *2,3,5,6-tetrahydro-7H-thiazolo[3,2-a]pyrimidin-7-one* **24**

50 mg of *Thiazolidin-2-imine* (0.49 mmole) was mixed with 50 µL of methyl acrylate (0.58 mmole) in 1 mL of EtOH, containing 10 µL of triethylamine. The mixture was refluxed for 6 hours. Then the solvent and volatile reagents were evaporated and the compound was recrystallized from EtOH. Product slowly hydrolyzes, when dissolved in water. **^{1}H NMR** (300 MHz, DMSO). Yield: 40 mg (0.256 mmole, 52%) δ = 3.83 (t, J=7.50 Hz , 2H), 3.52 (t, J=7.57 Hz, 2H), 3.36 (t, J=7.50 Hz, 2H), 2.42 (t, J=6.86 Hz, 2H), 2.04 (p, J=7.57 Hz, 2H). **^{13}C NMR** (75.4 MHz, DMSO) δ = 177.45, 175.25, 54.39, 43.35, 29.35, 26.84. **HRMS:** calcd. for $[M+H]^+$ $C_6H_9N_2S^+$ 157.0436; found 157.0434.

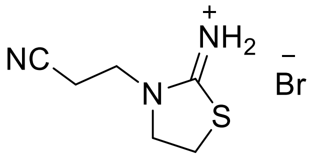

Synthesis of *3-(2-iminothiazolidin-3-yl)propanenitrile*

230 mg of *Thiazolidin-2-imine* (2.25 mmole) was mixed with 300 µL of acrylonitrile (4.6 mmole) in 2 mL of DMF. Triethylamine (70 µL, 0.5 mmole, 22 mol %) was added to the mixture and the reaction was heated to 70ºC. Solvent was evaporated and the product was recrystallized from EtOH as hydrobromide. Compound spontaneously decomposes, when dissolved in water. Yield: 310 mg (1.35 mmole, 60%). **^{1}H NMR** (300 MHz, DMSO). δ = 10.00 (s, 1H), 9.57 (s, 1H), 4.07 (t, J=7.50 Hz, 2H), 3.87 (t, J=6.86 Hz, 2H), 3.50 (t, J=7.57 Hz, 2H), 2.96 (t, J=7.57 Hz, 2H). **^{13}C NMR** (75.4 MHz, DMSO) δ = 170.82, 118.82, 54.82, 42.74, 28.38, 28.38. **HRMS:** cd. for $[M+H]^+$ $C_6H_{10}N_3S^+$ 156.0595; found 157.0595.

3. Single-crystal X-ray diffraction analysis of 16, 28, 26 and 24.

A single crystal was coated in Paratone oil (Hampton Research) and mounted on a MiTeGen loop. It was flash frozen in a liquid nitrogen stream of Oxford Cryostream. Diffraction data were measured at a low temperature of 100(2) K using MoKα λ = 0.71073 Å on a Bruker KappaApexII diffractometer for **16**, Rigaku Synergy-S for **24**, and using CuKα λ = 1.540184 Å on a Rigaku XtaLabPro diffractometer for **28** and **26**. The data were processed and reduced with the Bruker Apex3 Suite of programs for **16** and CrysAlis Pro for **28**, **26** and **24.**. The structure was solved by direct methods with SHELXT-2014/5 for **16**, SHELXT-2016/4 for **28** and SHELXL-2014/7 for **26** and **24**. All non-hydrogen atoms were refined anisotropically. Hydrogens on the amine group were located in the difference density electron density peaks and refined independently. All other hydrogens were placed in calculated positions and refined in riding mode. The crystal data and the structural refinement are summarized in **Supplementary Table 1**.

Supplementary Table 1. Crystal data and structural refinement

Crystals and symbols	**16**	**28**	**26**	**24**
Measurement name	ss4b	ss15b	ss18	ss11
CCDC	2033291	2042595	2062125	2063063
Crystal description	Colourless plate	Colourless plate	Colourless plate	Colourless chunk
Diffractometer	Bruker KappaApexII	Rigaku XtaLabPro	Rigaku XtaLabPro	Rigaku Synergy-S
Empirical formula	$C_4H_6N_2O_2S$	$C_{14}H_{20}N_6O_2S_2$	$C_6H_{11}BrN_4OS$	$C_6H_8N_2OS$
Formula weight (g/mol)	146.17	368.48	267.16	156.20
Temperature (K)	100	100	100	100
Wavelength (Å)	0.71073	1.540184	1.540184	0.71073
Crystal system	monoclinic	orthorhombic	monoclinic	orthorhombic
Space group	*P2₁*	*Pbca*	*P2₁/c*	*Pbca*
a (Å)	5.7610(3)	7.7662(5)	5.4934(2)	9.7101(3)
b (Å)	5.3472(2)	15.3500(5)	18.6929(5)	8.9145(3)
c (Å)	9.7354(4)	28.7054(15)	9.3041(3)	15.5027(5)
α,β,γ°	90,93.251(1),90	90,90,90	90,97.709(3),90	90,90,90
Volume (Å^3)	299.42(2)	3422.0(3)	946.78(5)	1341.92(8)
Z	2	8	4	8
Density calculated (Mg/m^3)	1.621	1.431	1.874	1.546
Absorption coefficient (mm^{-1})	0.458	3.006	7.737	0.404
F(000)	152	1552	536	656
Theta range for data collection (°)	3.5 to 36.36	5.9 to 53.13	4.7 to 74.5	2.6 to 27.5
Reflection collected (Unique)	7960(2908)	12262(1971)	8030(1929)	26622(1538)
R_{int}	0.0218	0.0537	0.0741	0.0729

Completeness %	99.4	99.0	99.7	100.0
Data/restraints/parameters	2908 /1/ 94	1971/0/227	1929/1/121	1538/0/91
Goodness-of-fit on F^2	1.075	1.045	1.093	1.221
Final R [I>2σ(I)]	R_1=0.0208<br>wR_2=0.0528	R_1=0.0398<br>wR_2=0.0960	R_1=0.0760<br>wR_2=0.1999	R_1=0.0312<br>wR_2=0.0720
R (all data)	R_1=0.0217<br>wR_2=0.0532	R_1=0.0517<br>wR_2=0.1024	R_1=0.0815<br>wR_2=0.2045	R_1=0.0488<br>wR_2=0.0948
Largest diff. peak and hole ($e^{.}$ $Å^{-3}$)	0.372 and -0.150	0.248 and -0.370	1.602 and -1.477	0.358 and -0.268
Flack parameter	0.028(17)	NA	NA	NA

4. Batch kinetic experiments

4.1. General protocol for the ^{1}H NMR kinetics experiments

The kinetics of the interaction between thiouronium salts and cysteamine (**1**) or its homologues (**9** and **10**) were monitored by NMR. Usually, we ran the experiment with a concentration of both compounds around 50 mM in phosphate buffer solution (1 M and pH 7.5 or 8). Thiouronium salt **8** exhibited very high activity; monitoring of the reaction progress in experiments where the starting concentrations of reactants were 50 mM was not possible by NMR. For measuring the k_L value for **8**, we used 1 mM solutions. All measurements were carried out at room temperature, and changes in concentrations of the reactants and products were monitored by integration of the characteristic signals in ^{1}H NMR spectra.

4.2. Fitting the rate constants

The model of direct ligation was used to determine the rate constants (k_L) of the reactions. Best-fitted parameters were obtained by global fitting the data from the kinetic measurements to the model. The model was input to the software *COPASI* and the fittings were performed using the build-in function of "*Parameter Estimation*". The applied algorithm for fitting was *Evolutionary–Programming* (number of generations, 2000; population size, 30).[9] The fitting results are shown in Fig. 1 to 6. The imperfection of the fitting might be partially associated with the catalytic effect of 2-mercaptoethylguanidine (**11**), which is probably a better leaving group than MESNA thiol or ethanethiol. The catalytic effect of thiols that are good leaving groups is a well-known phenomenon in native chemical ligation,[10] and it should be expected in the thiol-assisted formation of guanidines.

Model: Direct ligation between thiouronium salts and the homologues of cysteamine with a rate of k_L.

$$\text{ThioU} + \text{CSH} \xrightarrow{k_L} \text{GSH} + \text{RSH}$$

The dynamics of the model is described by the following differential equations (1) to (4):

$$\frac{d[ThioU]}{dt} = -k_L[ThioU][CSH] \ (1)$$

$$\frac{d[CSH]}{dt} = -k_L[ThioU][CSH] \ (2)$$

$$\frac{d[GSH]}{dt} = k_L[ThioU][CSH] \ (3),$$

$$\frac{d[RSH]}{dt} = k_L[ThioU][CSH] \ (4)$$

where $[ThioU]$ is the concentration of thiouronium salts, and $[CSH]$, $[GSH]$, and $[RSH]$ represent the concentration of cysteamine, guanidine thiol, and the released free thiol, respectively.

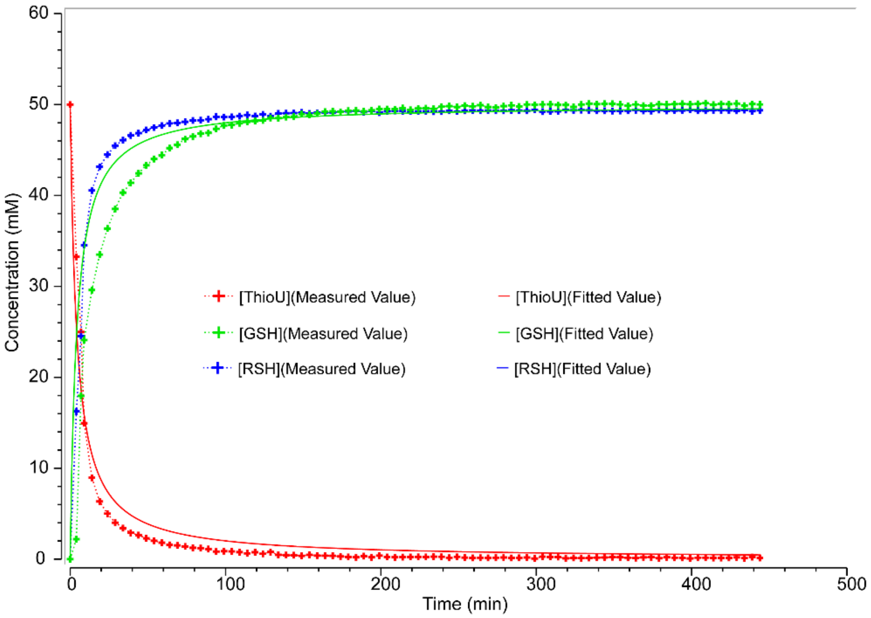

Supplementary Figure 1. Global fitting of the reaction between **3** (50 mM) and **1** (50 mM) in phosphate buffer pH 8. The best value of k_L (4.77×10^{-3} $min^{-1}mM^{-1}$) was obtained after parameter fitting.

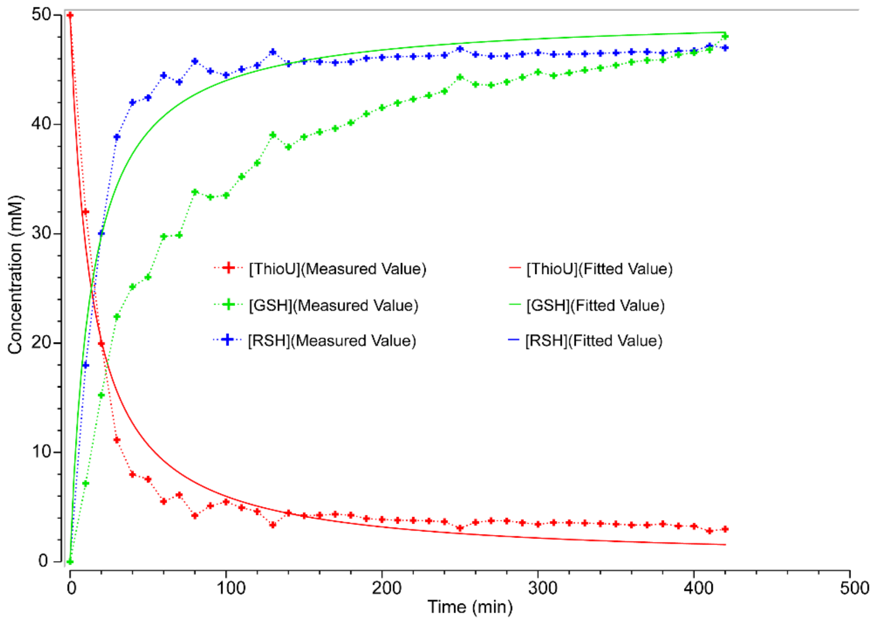

Supplementary Figure 2. Global fitting of the reaction between **3** (50 mM) and **9** (50 mM) in phosphate buffer pH 8. The best value of k_L (1.47×10^{-3} $min^{-1}mM^{-1}$) was obtained after parameter fitting.

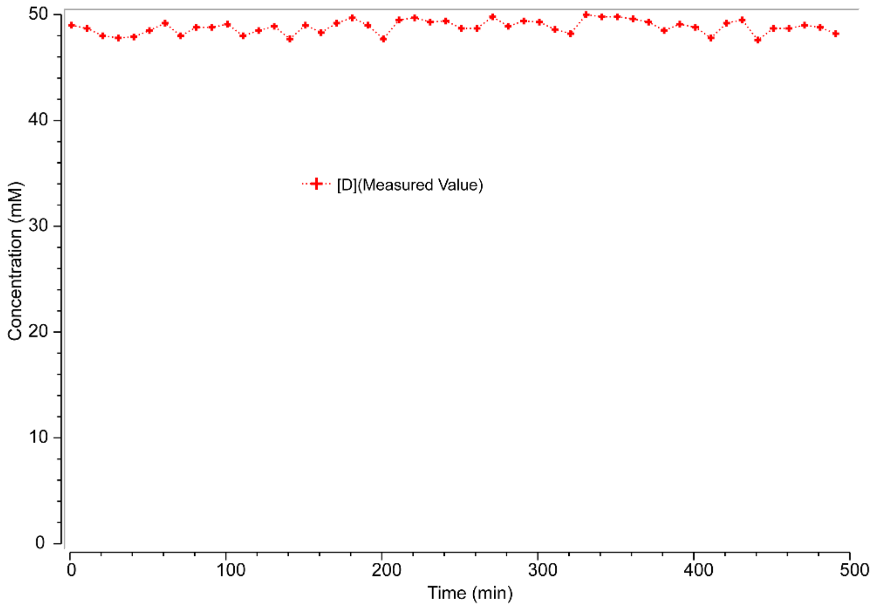

Supplementary Figure 3. Kinetic data of the reaction between **3** (50 mM) and **10** (50 mM) in phosphate buffer pH 8. The reaction did not proceed during the 500-minute period.

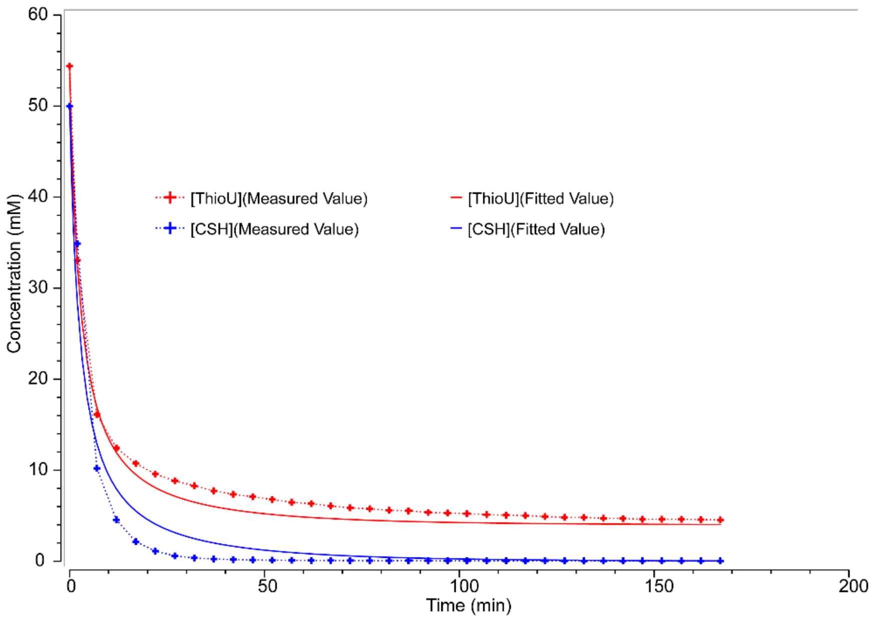

Supplementary Figure 4. Global fitting of the reaction between **4** (50 mM) and **1** (54 mM) in phosphate buffer pH 8. The best value of k_L (6.89×10^{-3} $min^{-1}mM^{-1}$) was obtained after parameter fitting.

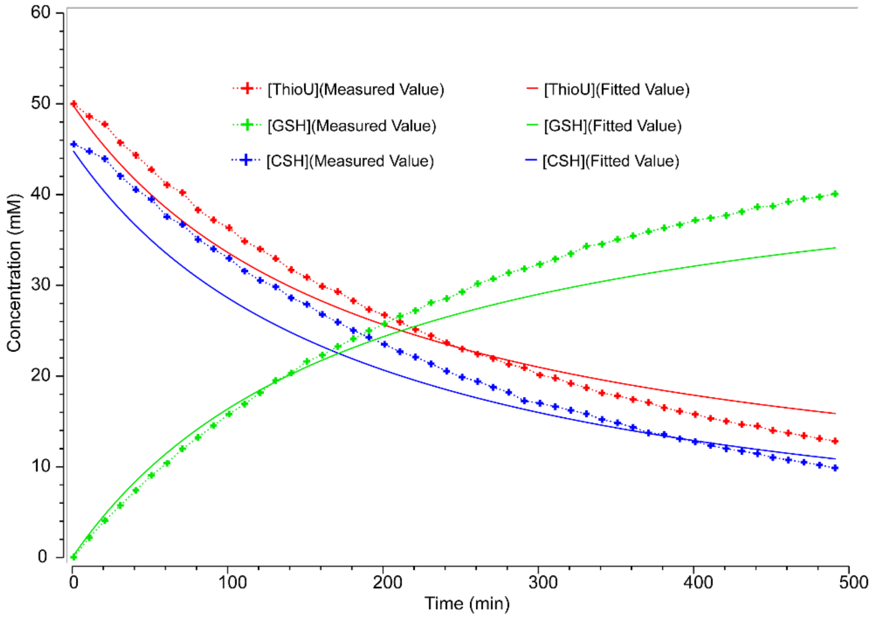

Supplementary Figure 5. Global fitting of the reaction between **5** (50 mM) and **1** (45 mM) in phosphate buffer pH 8. The best value of k_L (0.111×10^{-3} $min^{-1}mM^{-1}$) was obtained after parameter fitting.

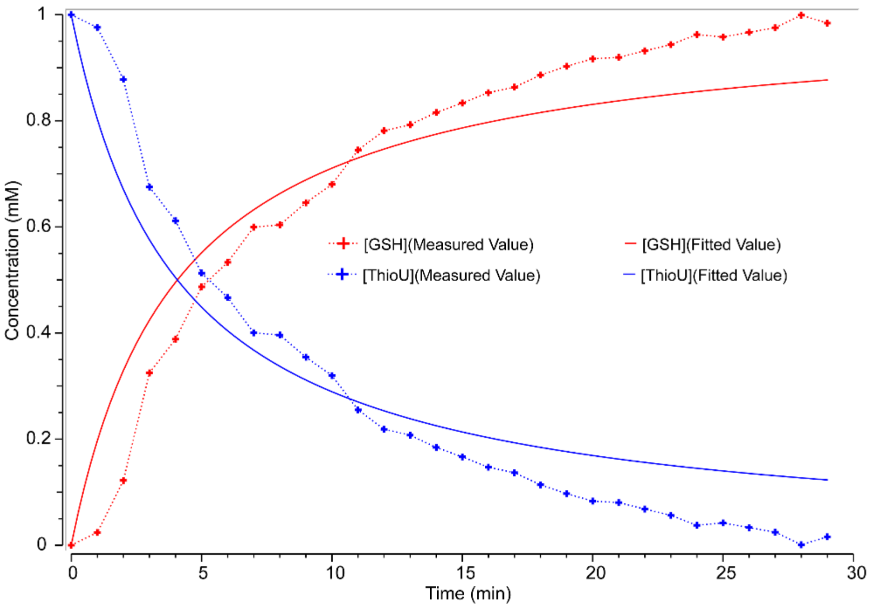

Supplementary Figure 6. Global fitting of the reaction between **8** (1 mM) and **1** (1 mM) in phosphate buffer pH 8. The best value of k_L (0.246 $min^{-1}mM^{-1}$) was obtained after parameter fitting.

Supplementary Table 2. Summary of the fitting of rate constants

Table S2| Ligation rate (kL) obtained after fitting

Substrate	Value ($M^{-1}min^{-1}$)	Standard Deviation	Value ($mM^{-1}min^{-1}$)	Standard Deviation
**3+1**	4.77143	1.02E-01	0.00477143	1.02E-04
**3+9**	1.47022	4.69E-02	0.00147022	4.69E-05
**3+10**	0	0.00E+00	0	
**4+1**	6.88852	2.42E-01	0.00688852	0.000242054
**5+1**	0.111158	1.67E-03	1.11E-04	1.67E-06
**8+1**	245.683	1.14E+01	0.245683	0.0114272

4.3. Batch experiments showing a pulse

To estimate the experimental parameters for oscillations in flow, we performed the pulse experiments under batch conditions and fitted the experimental data with the three-variable model of the oscillator (see the modeling section).

We used the following experimental protocol for the batch puls experiments. A freshly prepared solution of Ellman's reagent 2 mM in phosphate buffer solution (pH = 7.0; 200 mM) was used to fill about fifty UV-vis cuvettes with 2 ml of solution in each cuvette. Next, 10 μL aliquots of the reaction mixture were taken every minute and mixed with Ellman's solution. The absorbance at 412 nm was measured by a UV-Vis spectrometer and converted to the total concentration of thiols.

The results of these experiments are presented in the following two Supplementary Figures:

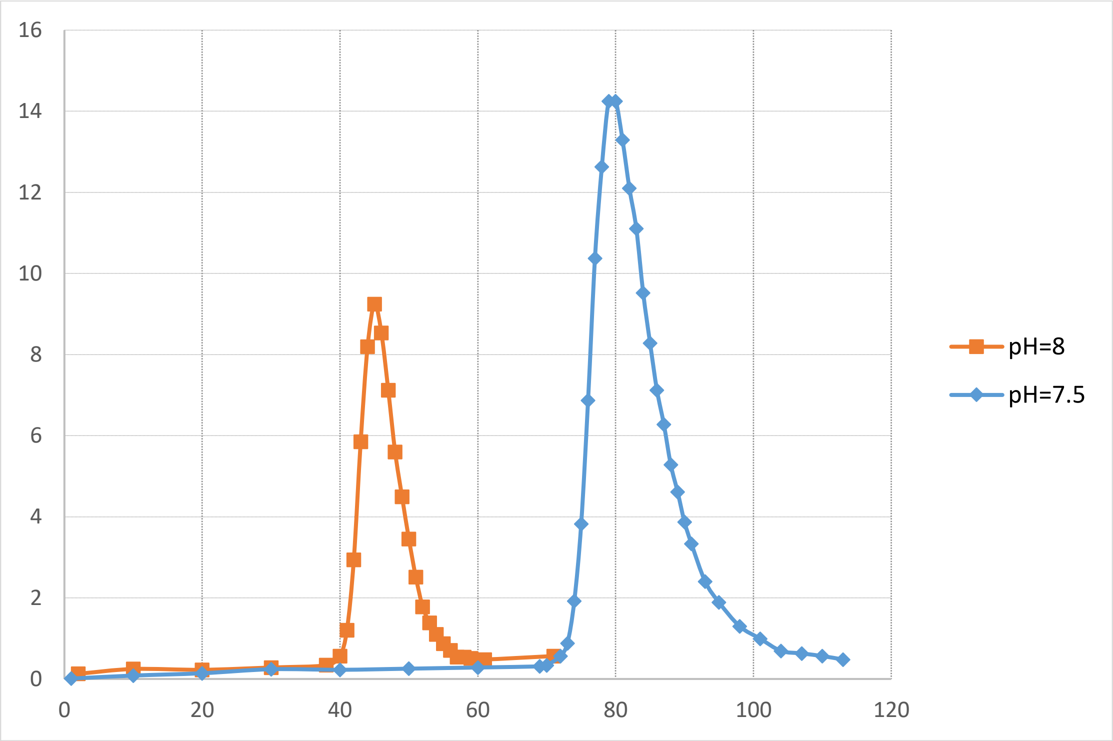

Supplementary Figure 7. Experiments showing a pulse under batch conditions. Blue curve, experimental conditions: H_2O, 1 M phosphate buffer pH 7.5, 25 ^oC; [**8**] = 50 mM; [**17**] = 50 mM; [Acrylamide] = 282 mM; [Maleimide] = 7.66 mM; orange curve, experimental conditions: H_2O, 1 M phosphate buffer pH 8, 25 ^oC; [**8**] = 50 mM; [**17**] = 50 mM; [Acrylamide] = 282 mM; [Maleimide] = 7.66 mM.

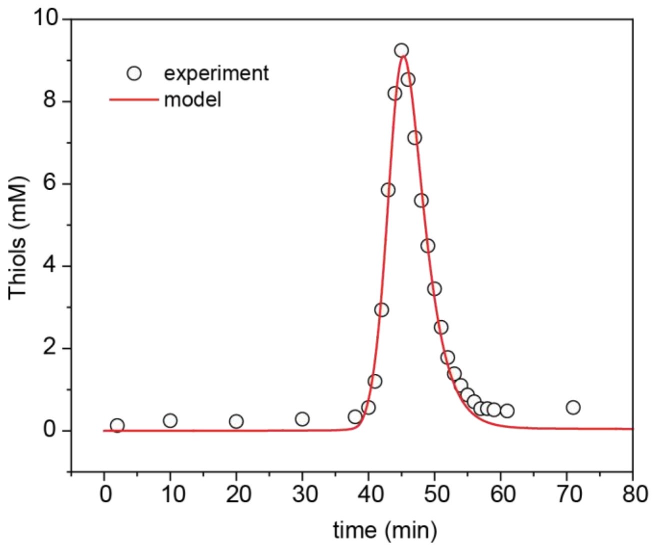

Supplementary Figure 8. Fitting of the experimental data for the pulse with the three-variable model. Experimental conditions: H_2O, 1 M phosphate buffer pH 8, 25 ^oC; [**8**] = 50 mM; [**17**] = 50 mM; [Acrylamide] = 282 mM; [Maleimide] = 7.66 mM. Model parameters obtained by fitting the experimental data are as follows: k_1 = 0.507 $s^{-1}M^{-1}$, k_2 = 300 $s^{-1}M^{-1}$, k_3 = 0.00932 s^{-1}, and k_4 = $7.09 \cdot 10^{-5}$ s^{-1}.

5. Supplementary discussion of the one-pot cyclization reactions

5.1 Formation of bicyclic products via tandem cyclization of the guanidines formed in the autocatalytic reaction.

Reaction 1

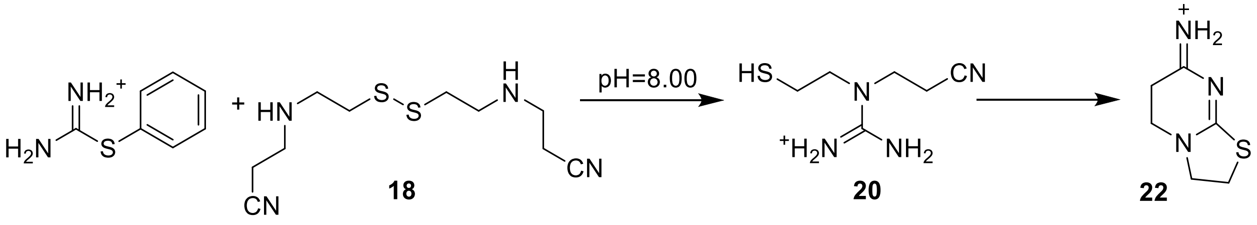

16.2 mg (70 mmol) of phenylthiouronium bromide was mixed with 20.5 mg (70 mmol) of nitrile in 1 mL of phosphate buffer (pH=8.00) in D_2O. The reaction was continuously monitored by recording ^{1}H NMR spectrum every 10 min for 8 hours.

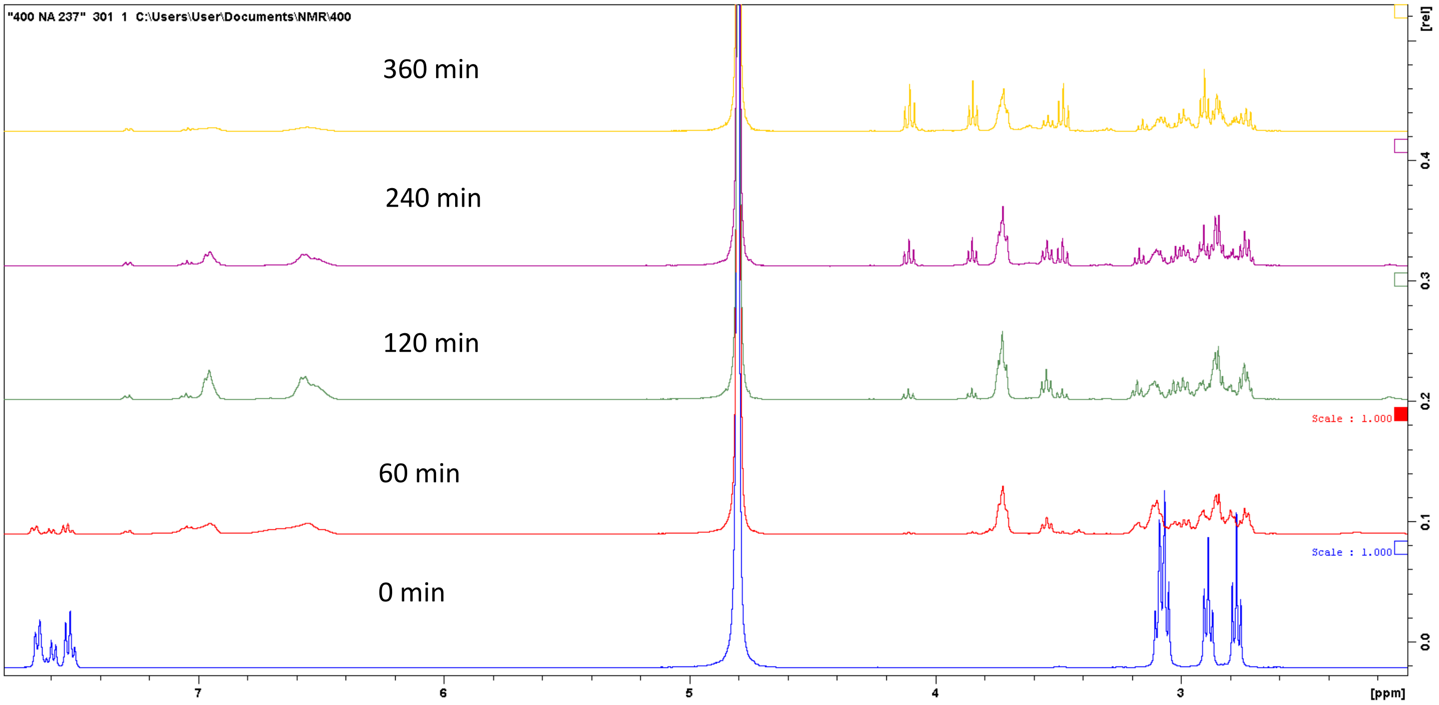

Supplementary Figure 9. ^{1}H NMR (400 MHz, D_2O) spectra, representing the progress of the **reaction 1**. Two consequent processes occur during the reaction. First, the initial growth of the overlapped peaks at 3.80-3.68 ppm, which we assign to CH_2 protons adjacent to the guanidine functional group in the intermediate. After

initial fast growth, those peaks decrease over time. The peaks at 4.10, 3.85, 3.48, 2.90 ppm were assigned to the final product. They grow until the end of the reaction.

To gain the further information about the product of the reaction we recorded the HPLC-MS spectrum from the reaction mixture. After five hours of reaction we observed the emergence of the peak with m/z=156.2, corresponding to the bicyclic amidine **22** ($[M+H]^+$) and peaks with m/z = 173.2, 172.2, corresponding to the thiol **20** ($[M+H]^+$) and its disulfide ($[M+2H]^{2+}$) respectively.

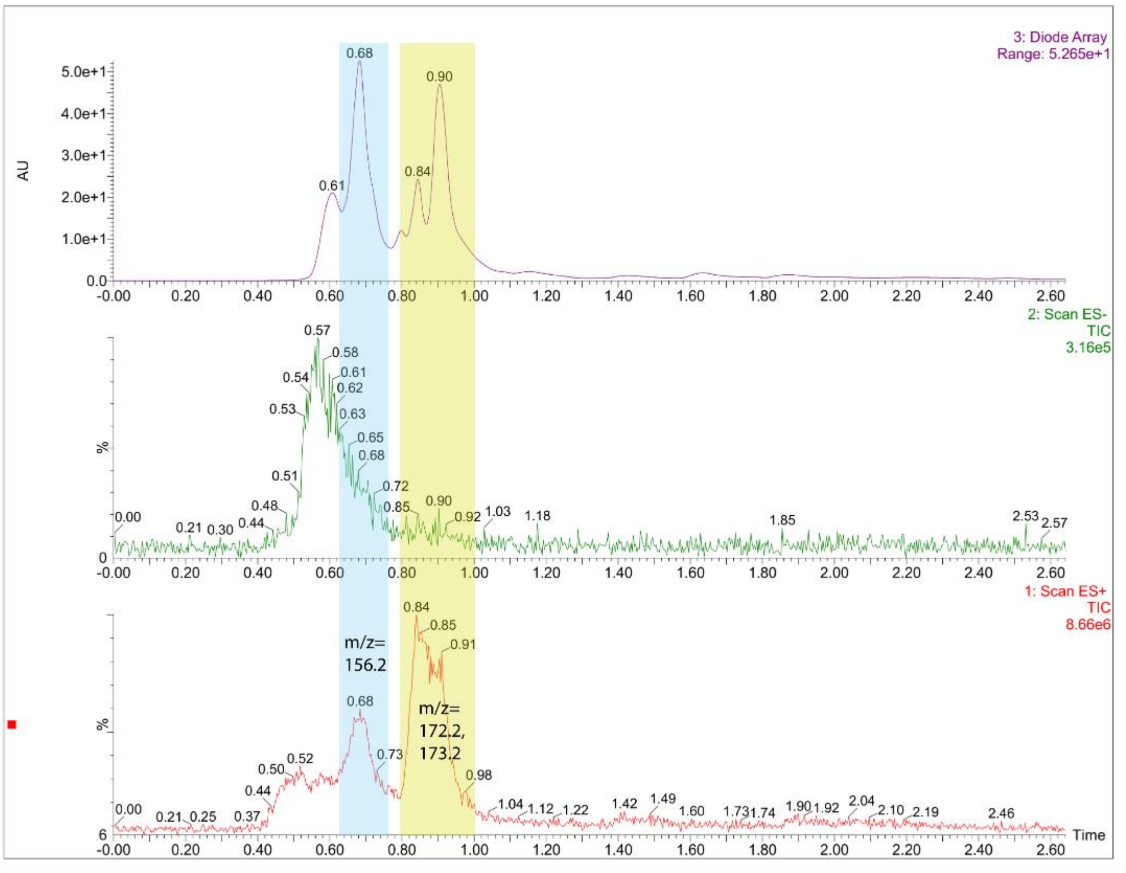

Supplementary Figure 10. LCMS spectrum obtained after 5 h of **reaction 1**.

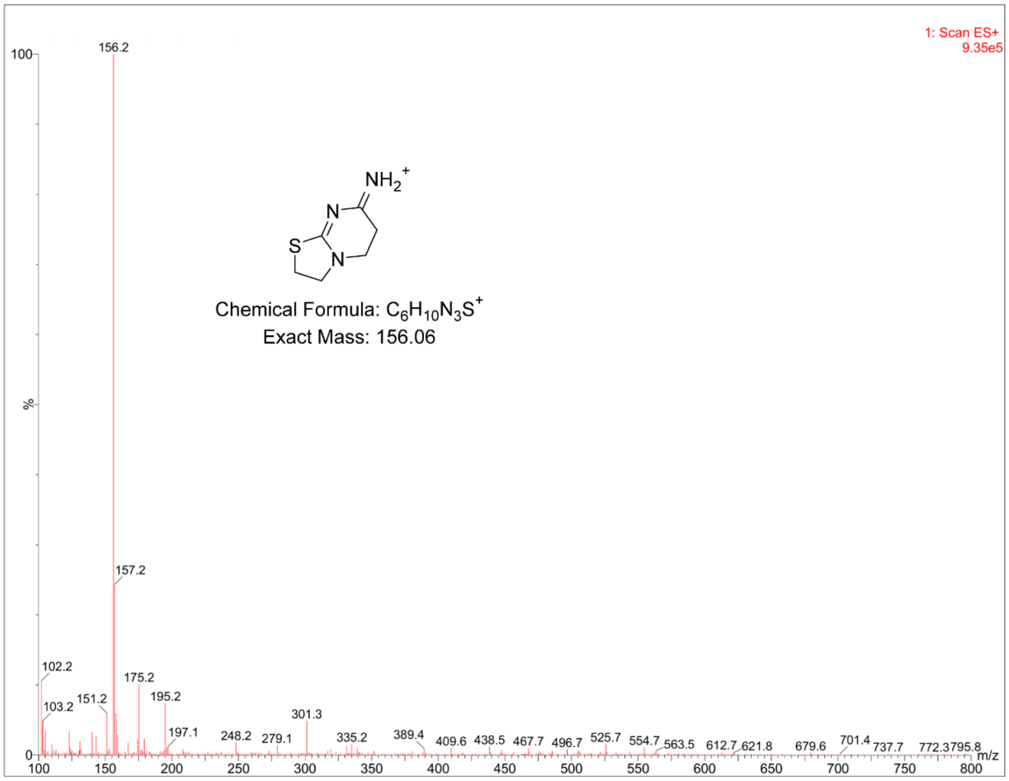

Supplementary Figure 11. Mass-spectrum corresponding to the blue region in LCMS chromatogram (Supplementary Figure 10).

After 24 hours of monitoring, peak m/z=173.2 disappeared and peak m/z=156.2 remained.

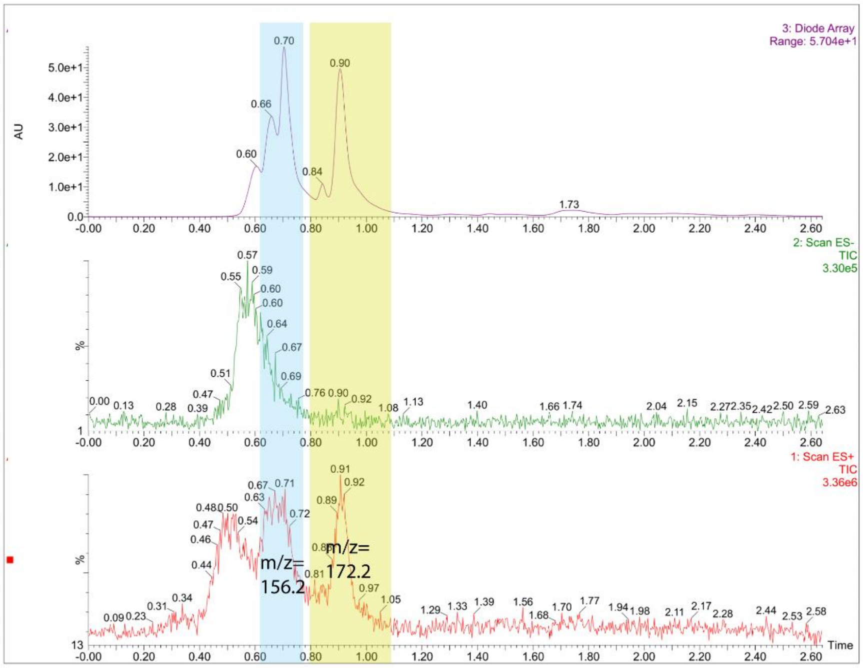

Supplementary Figure 12. LCMS spectrum obtained after 24 h of **reaction 1**.

To better analyze structures of the products, we scaled up the **Reaction 1.** 232 mg (1 mmole) of phenylthiouronium bromide and 165 mg (0.5 mmole) of dinitrile were mixed in 5 mL of 1M phosphate buffer (pH=8.00). The mixture was left to stir overnight. The precipitate of thiophenol disulfide was filtered and the mixture was concentrated until 1/10 of the volume. Then hot EtOH was added to extract the product from inorganic salts. The presence of the target compound in the ethanol extract was verified using LCMS. After evaporation of ethanol most of the signal of m/z=156 shortly converts converted to m/z=157. After the flash column chromatography ($CHCl_3$/MeOH=15/1) small amount (15 mg) of the compound **24**, contaminated with its methanolysis product, was collected.

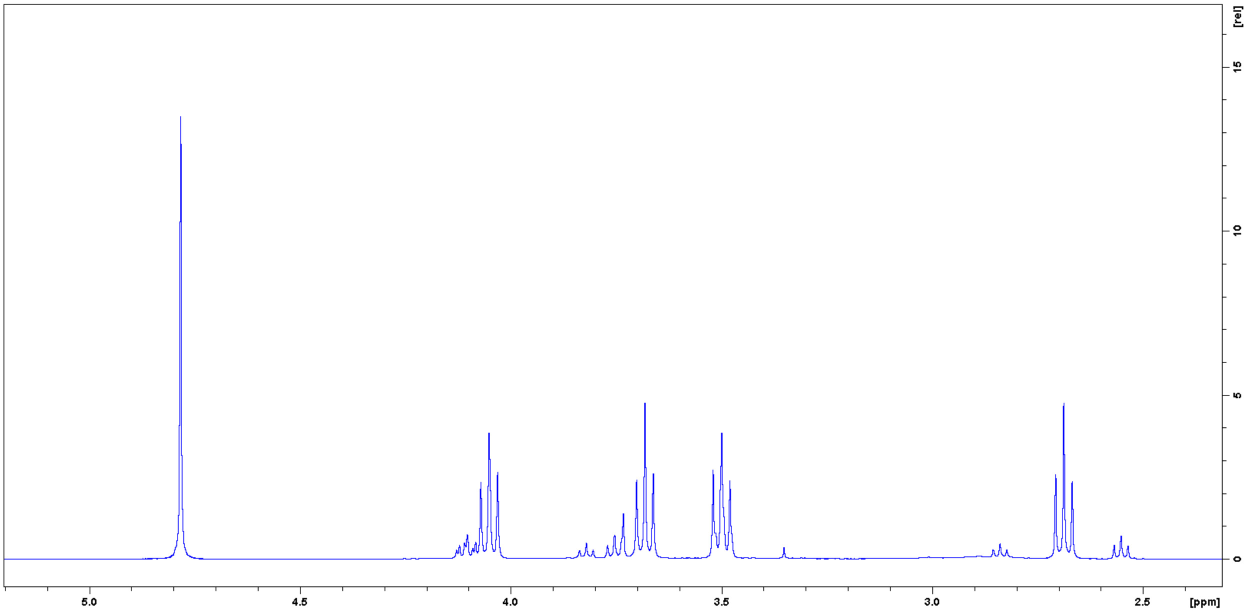

Supplementary Figure 13. ^{1}H NMR (400 MHz, D_2O) of the compound **24**, separated from the scaled up reaction mixture. Small peaks supposedly correspond to the product of methanolysis of the target compound.

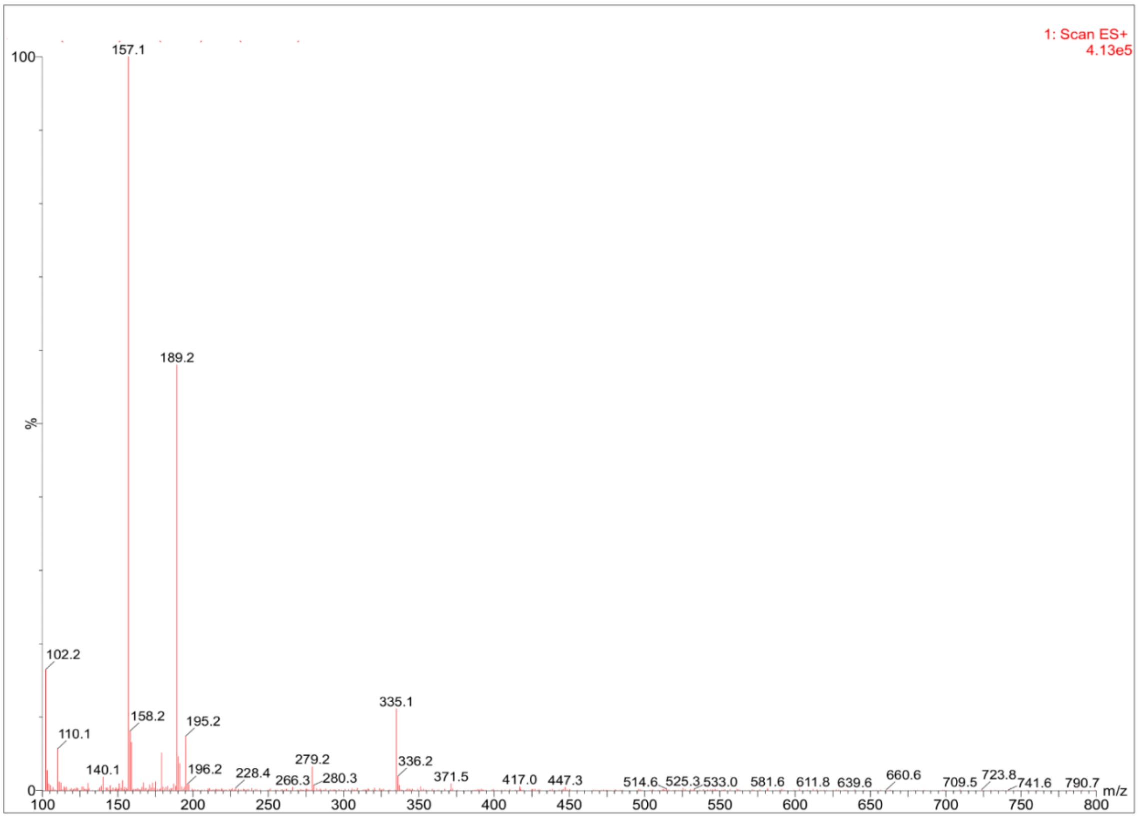

Supplementary Figure 14. LRMS spectrum of the compound **24** separated from the scaled up reaction mixture. We consider that the peak 189.2 corresponds to the target compound adduct with MeOH.

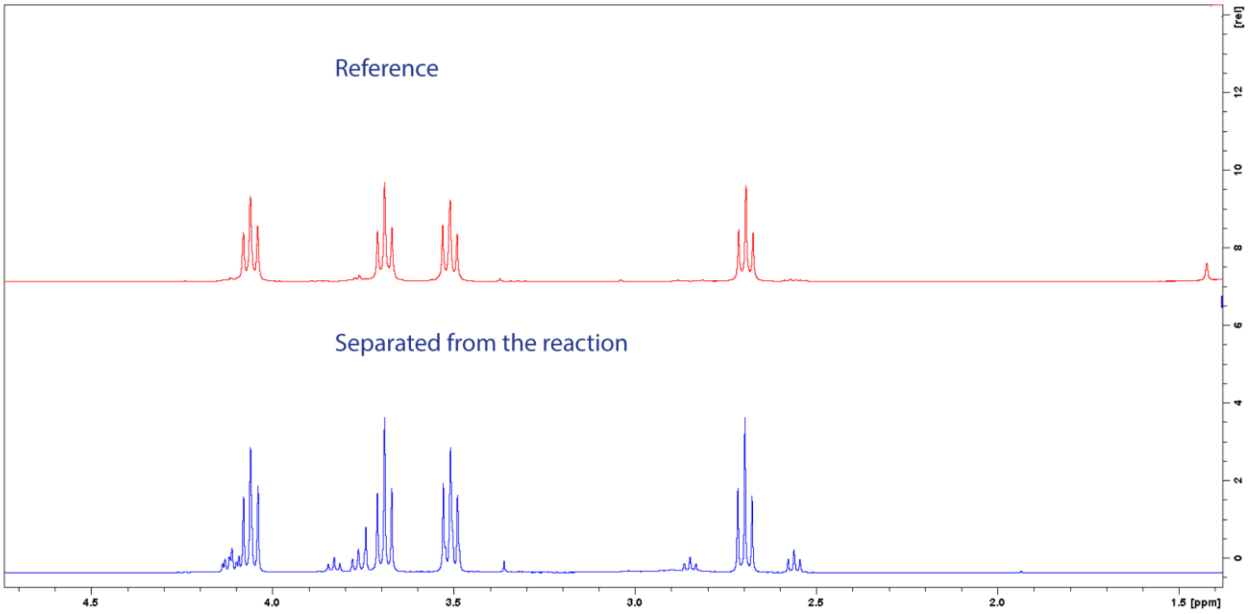

Supplementary Figure 15. Comparison of ^{1}H NMR (400 MHz, D_2O) spectra of the reference **24** synthesized by independent method and **24** separated from the reaction mixture.

Hence, we ensured that peak m/z=157 belongs to the bicyclic amide **24**. To get an additional evidence that the compound with m/z=156 is bicyclic amidine **22**, we synthesized 3-(2-iminothiazolidin-3-yl)propanenitrile. This nitrile after being dissolved in potassium phosphate buffer pH=8.00 (**reaction 2**). immediately decomposed, producing some amount of a compound whose spectrum resembles the one from bicylic amide **24.**

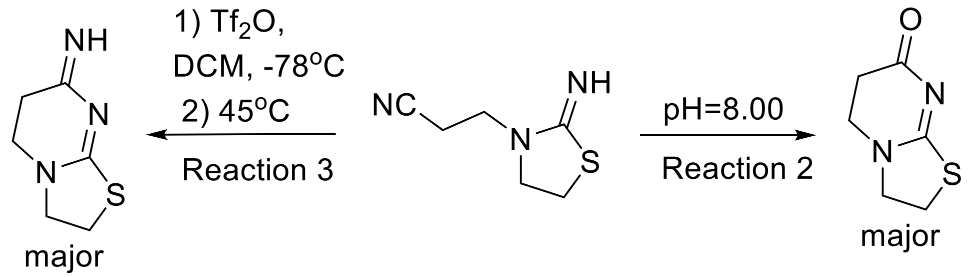

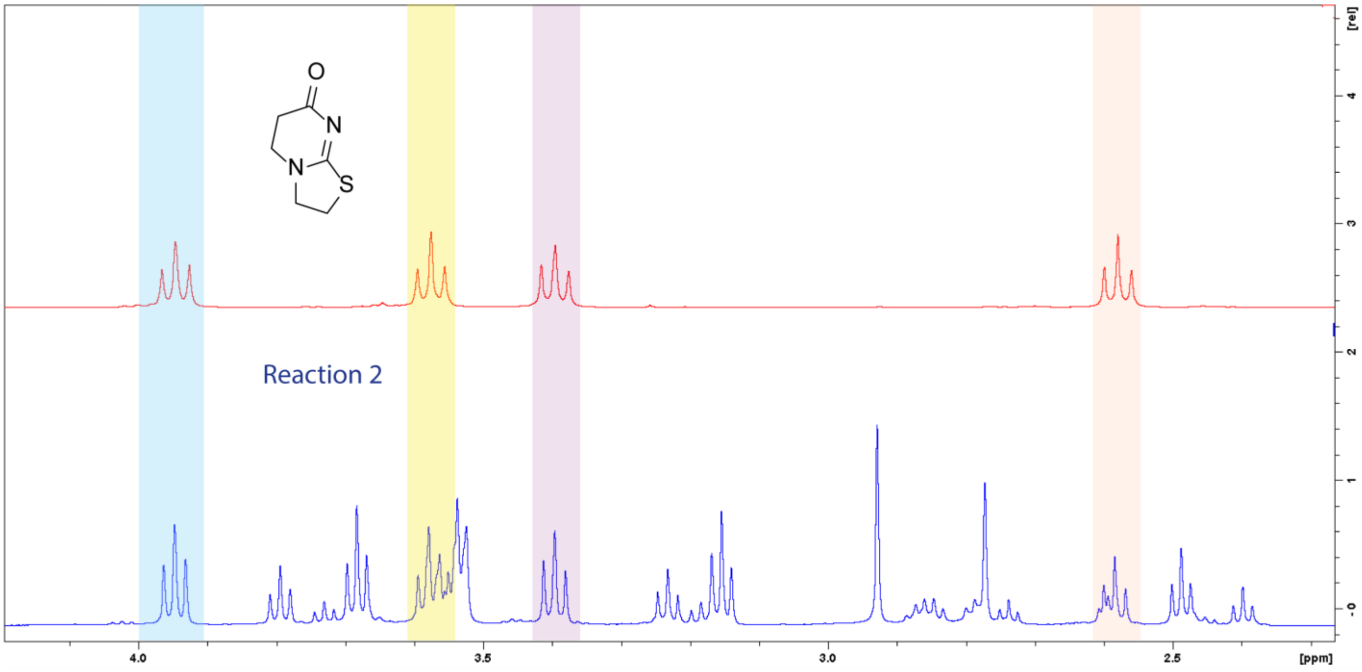

Supplementary Figure 16. Comparison of ^{1}H NMR (400 MHz, 500 MHz, respectively, D_2O, pH=8.00) of the bicylic amide **24** and the mixture from the **reaction 2**. Highlighted peaks confirm the presence of amide **24** in reaction mixture **2.**

To obtain the reference sample of bicyclic amidine **22** we conducted the following reaction (**Reaction 3**). To a solution of 3-(2-iminothiazolidin-3-yl)propanenitrile hydrobromide (50 mg, 0.32 mmole) in dry DCM was added triflic anhydride (100 uL) at -78°C, under inert atmosphere. The reaction was kept at this temperature for 10 minutes and then slowly heated to 0°C. After 10 minutes at this temperature, the reaction mixture was

transferred to an oil bath and heated to 45°C. Then solvents and volatile reagents were evaporated on the oil pump and ^{1}H NMR was recorded.

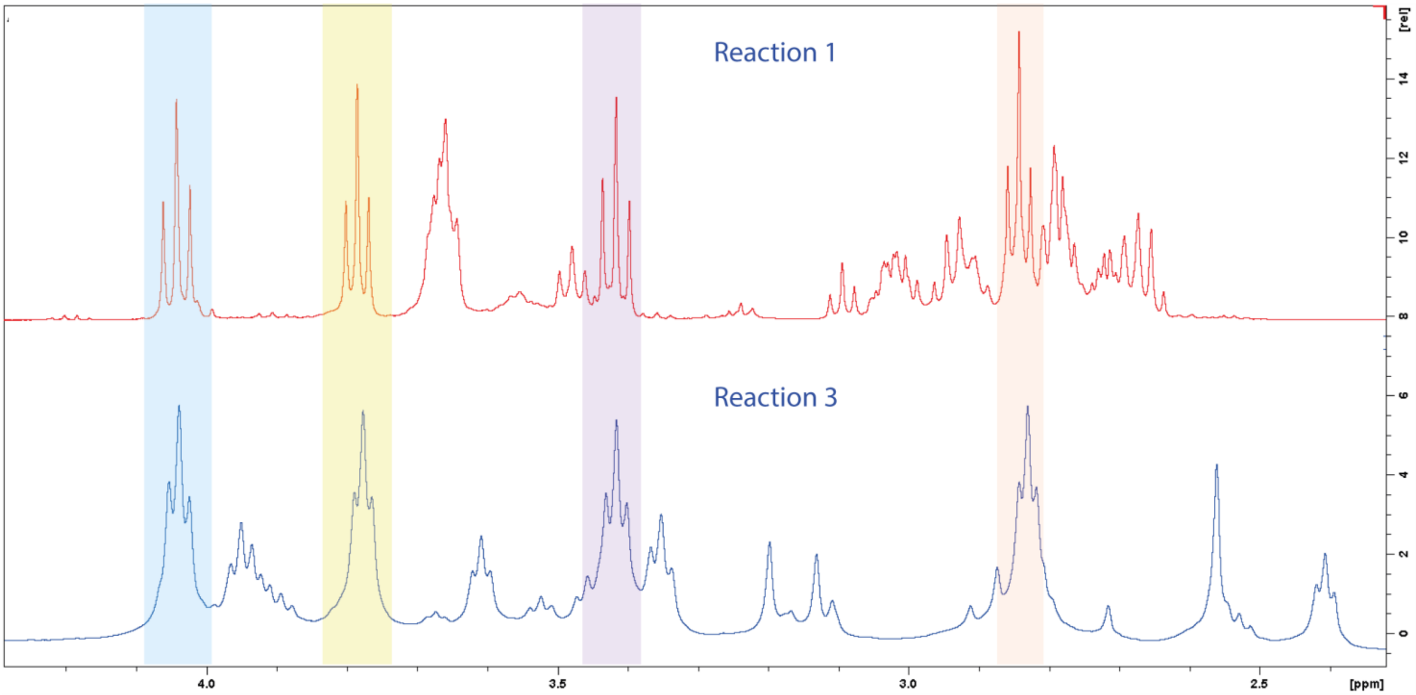

Supplementary Figure 17. Comparison of 1H NMR (400 MHz, 500 MHz, respectively, D_2O, pH=8.00) of the product mixture from the **reaction 1** and **reaction 3**. Similarity of highlighted peaks confirms the presence of amidine **22** in both reactions.

The results of these studies can be summarized in three conclusions:

1) One of the products of the **reaction 1** is bicyclic amide **24**. It can be separated from the reaction mixture using silica column chromatography. Its spectrum showed complete correspondence with **24** that we synthetized by the alternative procedure.
2) Product with m/z=156 is bicyclic amidine **22**. Its ^{1}H NMR spectrum matched with the amidine **22** obtained from the **reaction 3** as reference. This product, however, is unstable during workup and converts to the bicyclic amide **24**.
3) In buffer, thiol **20** undergoes spontaneous cyclization into amidine **22**, which then leads to the production of amide **24**.

Those conclusions can be summarized into the following mechanism:

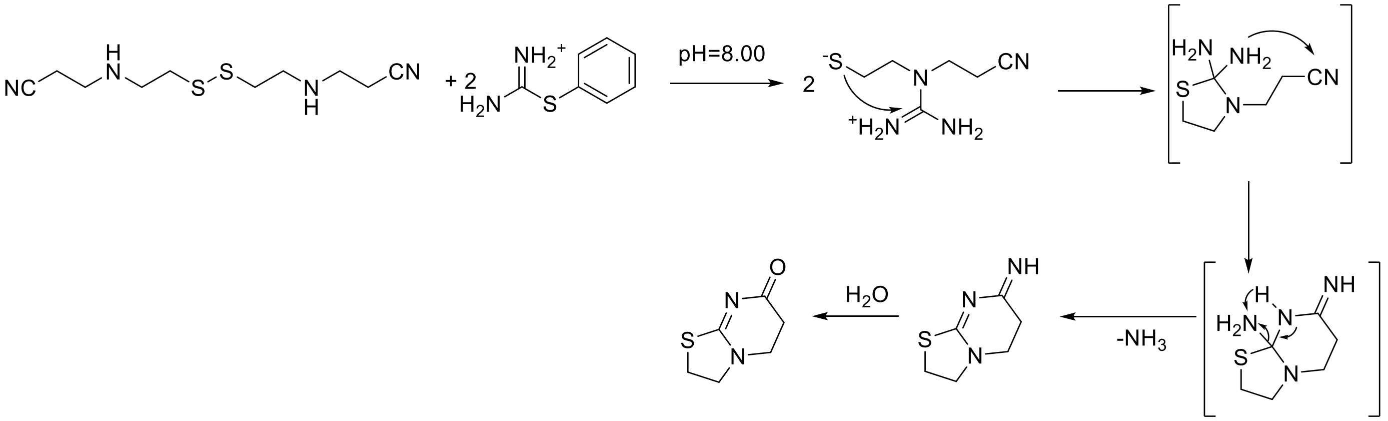

Supplementary Figure 18. Suggested mechanism for the formation of bicyclic products from the **reaction 1**

To evaluate the ability of nitrile **19**, homologous to the nitrile **18**, to produce the bicyclic products from the similar reaction pathway we studied the **reaction 4**

Reaction 4

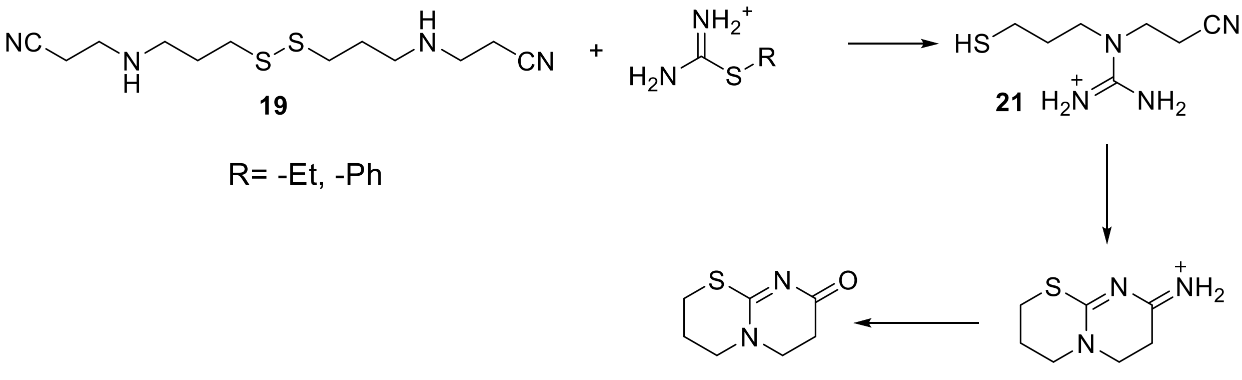

16.2 mg (70 mmol) of phenylthiouronium bromide was mixed with 27.8 mg (70 mmol) of dihydrobromide of **19** in 1 mL of phosphate buffer (pH=8.00) in D_2O. The reaction was continuously monitored by recording ^{1}H NMR spectrum every 20 min for 8 hours.

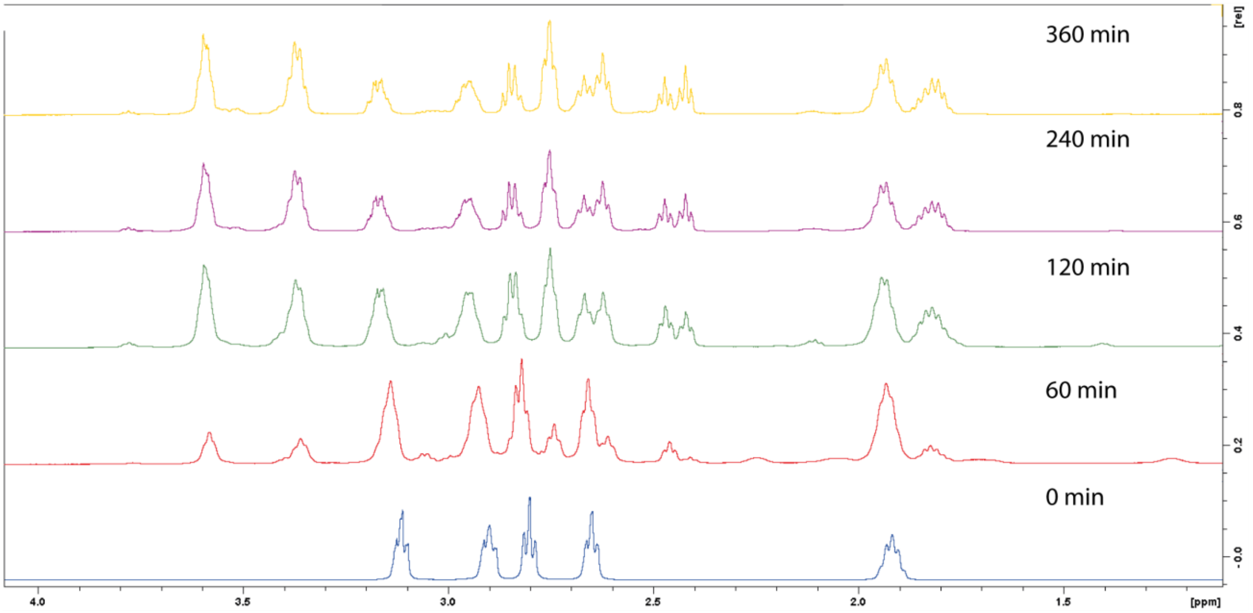

Supplementary Figure 19. ^{1}H NMR (500 MHz, D_2O, pH=8.00) spectra, representing the progress of the **reaction 4** (R=-Ph). Growth of adjacent to guanidine CH_2 peaks is clearly visible (3.58 ppm, 3.36 ppm). No cyclization product was observed.

According to our prior knowledge, the ligation of thiouronium salts with $(H_2N\text{-}(CH_2)_3\text{-}S)_2$ is much slower than one with cystamine (see Figure 2, main text). Here this observation is also supported, since guanidine formation is slower than in the **reaction 1.** No cyclization product was observed in the kinetic experiment as expected from the slower intramolecular cyclization of 3-mercaptopropylguanidines than of 2-mercaptoethylguanidines. However, increasing concentrations of reactants and the reaction time indeed has led to the production of bicyclic products.

Phenylthiuronium bromide (47 mg, 0.25 mmol) and disulfide **19** (45 mg, 0.125 mmol) were mixed in 1 mL of PB (1.5 M, pH 8). After 12 hours, LCMS chromatogram was recorded using SILEC Primeser 500 column.

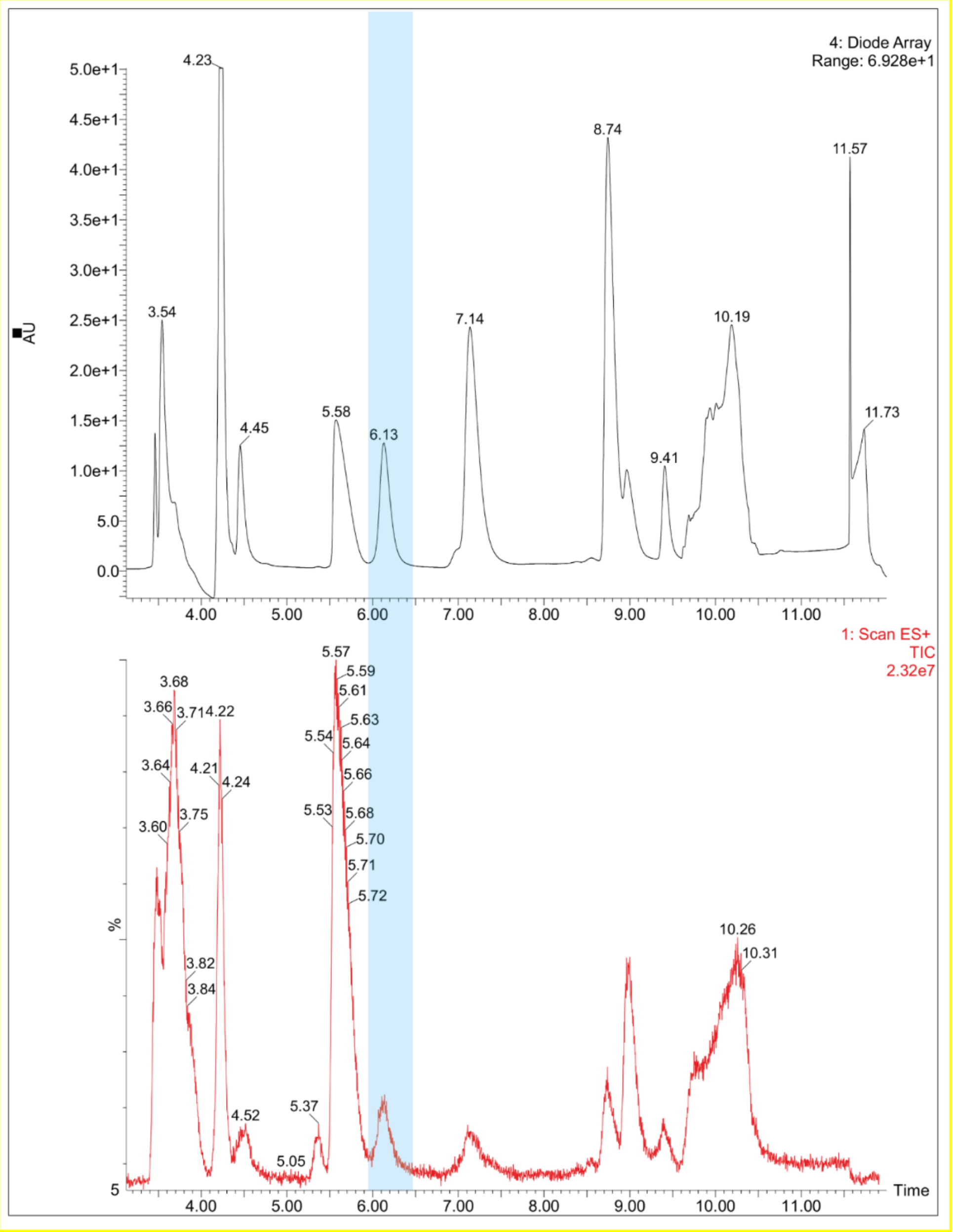

Supplementary Figure 20. LCMS chromatogram, representing the products of the **reaction 4** (R=Ph), under modified conditions. Highlighted peak has m/z = 170 (see Supplementary Figure below for MS data) and the retention time of 6.1 min falls to the expected region based on the 4.4 min retention time for **22** in the identical conditions.

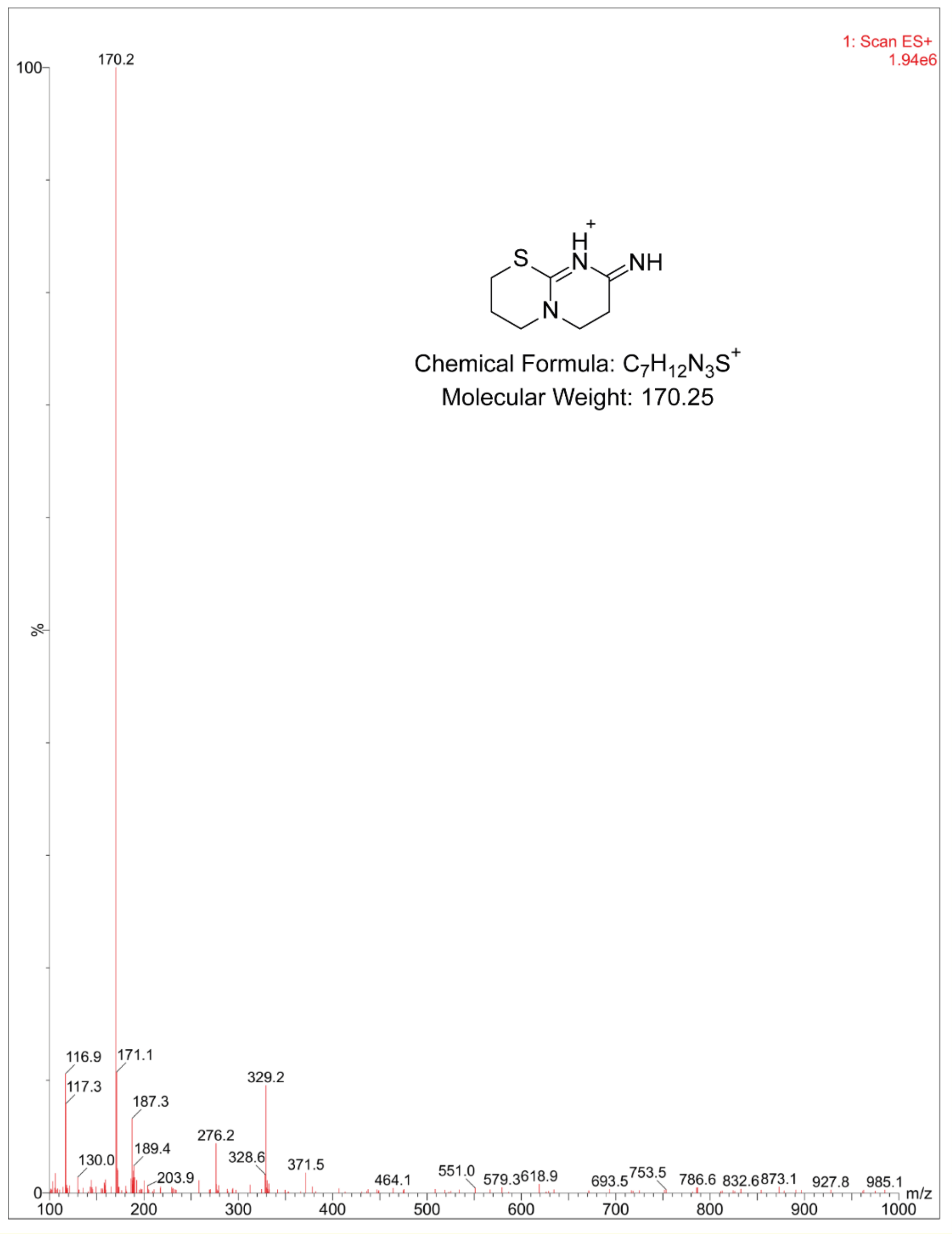

Supplementary Figure 21. MS spectrum of the region, highlighted in blue on the chromatogram (Fig. 20). Peak with m/z=170.2 matches with the one, predicted for the bicyclic amidine **23**.

Further increase of the reaction time results in the bicyclic amide being the major product. Ethylthiuronium bromide (46.5 mg, 0.25 mmol) and disulfide **19** (45 mg, 0.1 mmol) were mixed in 1 mL H_2O, and Na_2HPO_4 (146 mg) was added. After 48 hours, ^{1}H NMR, ^{13}C NMR, and LCMS chromatogram were recorded.

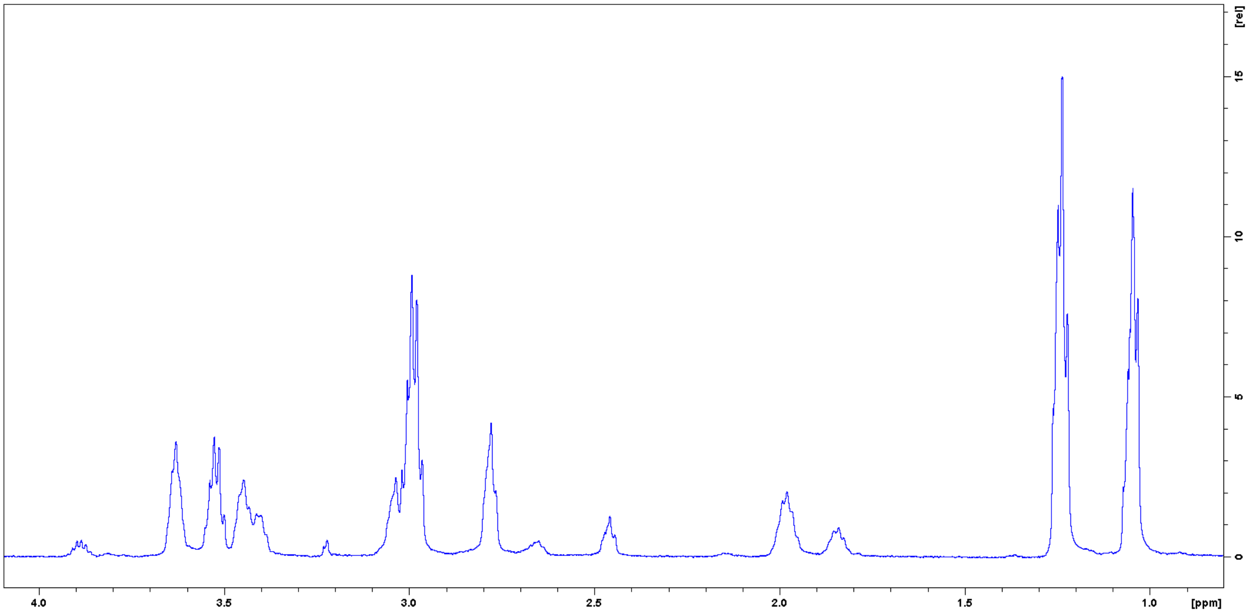

Supplementary Figure 22. ^{1}H NMR (500 MHz, H_2O, water suppression) spectrum, showing the products of **reaction 4** (R=-Et)**,** under modified condition. Two main product, resulting from disulfide **19** are well observable

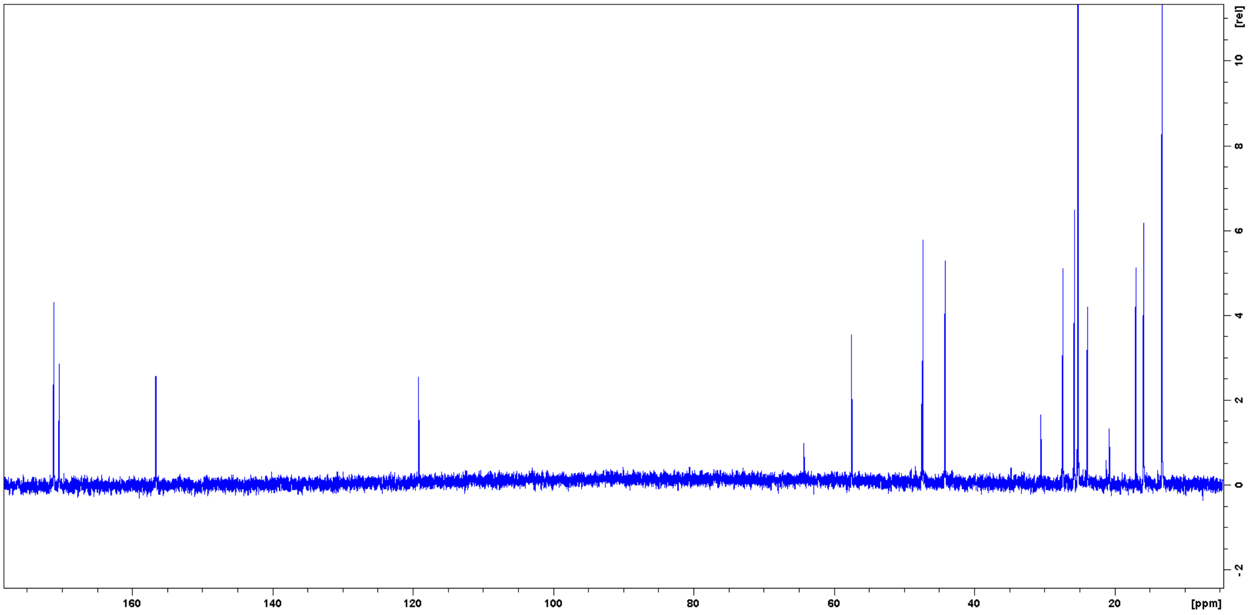

Supplementary Figure 23. ^{13}C NMR (125 MHz, H_2O) spectrum, representing the products of the **reaction 4** (R=-Et), under modified conditions. Three quaternary carbons in the region of carbonyl carbons and just one in the region of nitrile stand as an evidence for the nitrile cyclization.

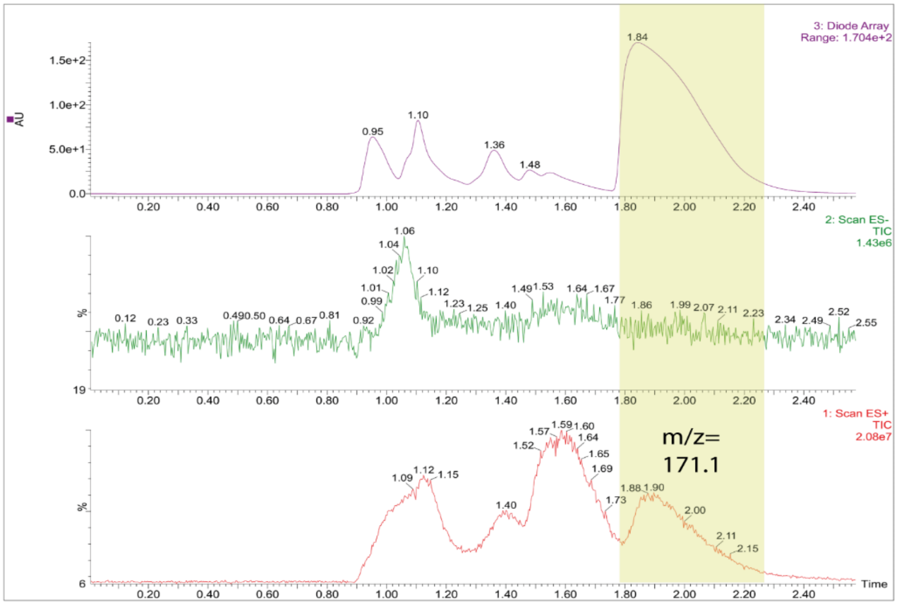

Supplementary Figure 24. LCMS chromatogram, representing the products of the **reaction 4** (R=-Et), under modified conditions. Peak with m/z=171.1 matches with the one, predicted for the bicyclic amide.

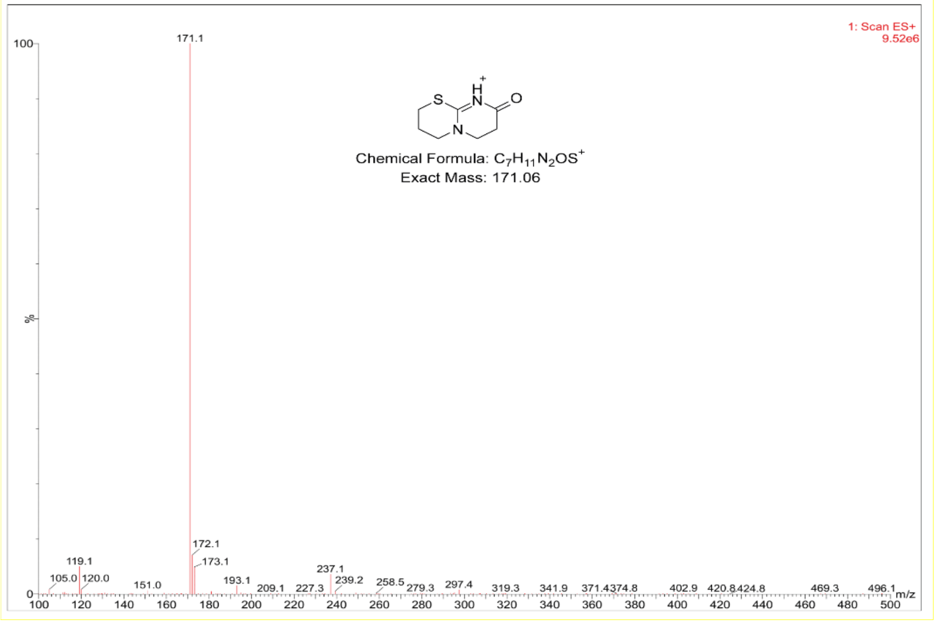

Supplementary Figure 25. MS spectrum of the region, highlighted in yellow on the chromatogram (Fig. 24), representing the dominating product of the **reaction 4** (R=-Et), under modified conditions. Peak with m/z=171.1 matches with the one, predicted for the bicyclic amide.

Based on these data, we conclude that **21** first cyclizes into bicyclic amidine **23** which then hydrolyses to bicyclic amide. This finding let us to assume that the **reaction 4** proceeds via the analogous mechanism to the **reaction 1.**

5.2 Oxidation of the dihydropyrimidine derivative 24 to pyrimidine derivative 25.

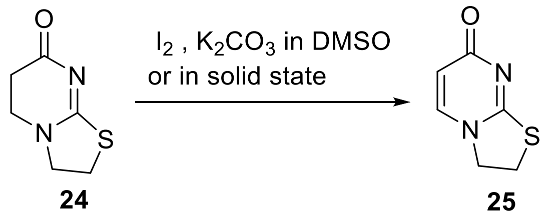

40 mg (0.12 mmole) of disulfide **18** hydrochloride and 56 mg (0.24 mmole) of phenylthiouronium salt hydrobromide were dissolved in 1ml of PB (pH=8, 1000mM) and heated to 60°C under argon atmosphere for 40 hours. Then solution was filtered to get rid of phenyldisulfide and water was evaporated under vacuum. Slightly wet mix of buffer salts and organic compounds were mixed with 100 mg of iodine and heated to 130° C for 30 minute, then mixture was treated with DMSO d^6 and NMR spectra was recorded. The reference NMR spectrum was obtained by heating pure **24** with iodine and K_2CO_3 in DMSO.

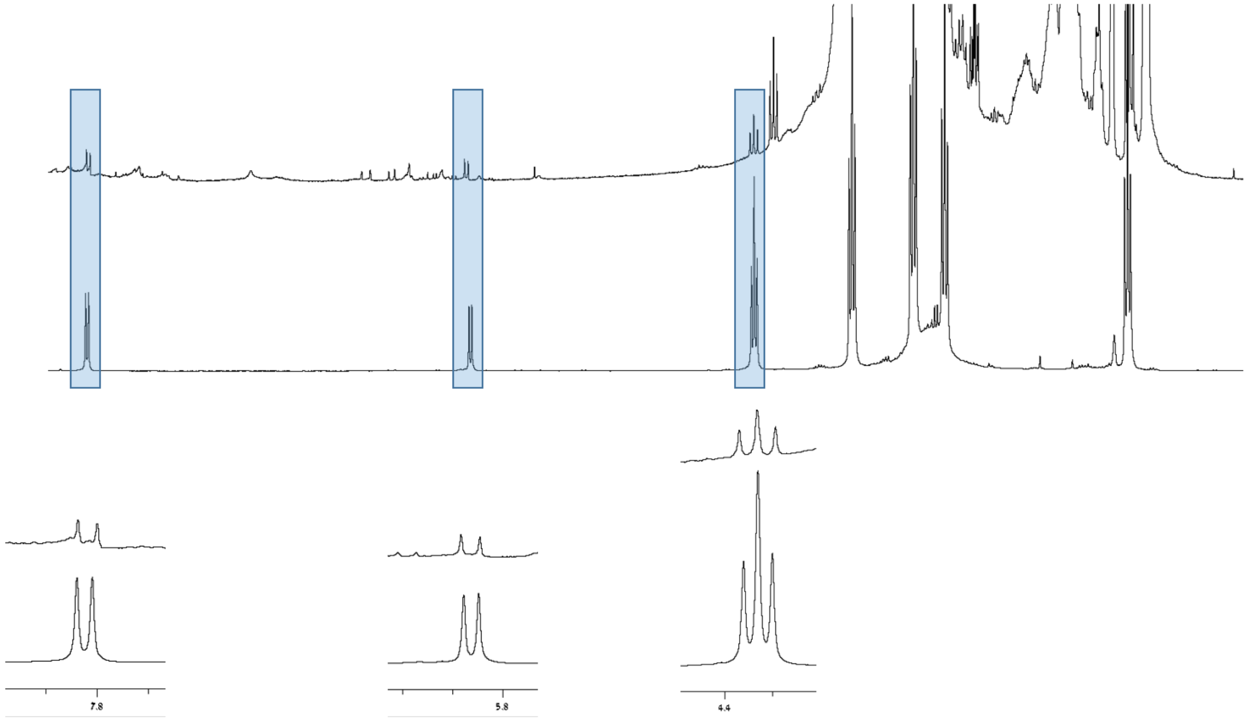

Supplementary Figure 26. The comparison of the ^{1}H NMR spectrum of the one-pot mixture and the reference ^{1}H NMR spectrum containing **25** and **24**. The inserts highlight the characteristic signals of **25** at 7.81, 5.83, and 4.37 ppm.

5.3 One-pot formation of the aminopyrimidine derivative 26.

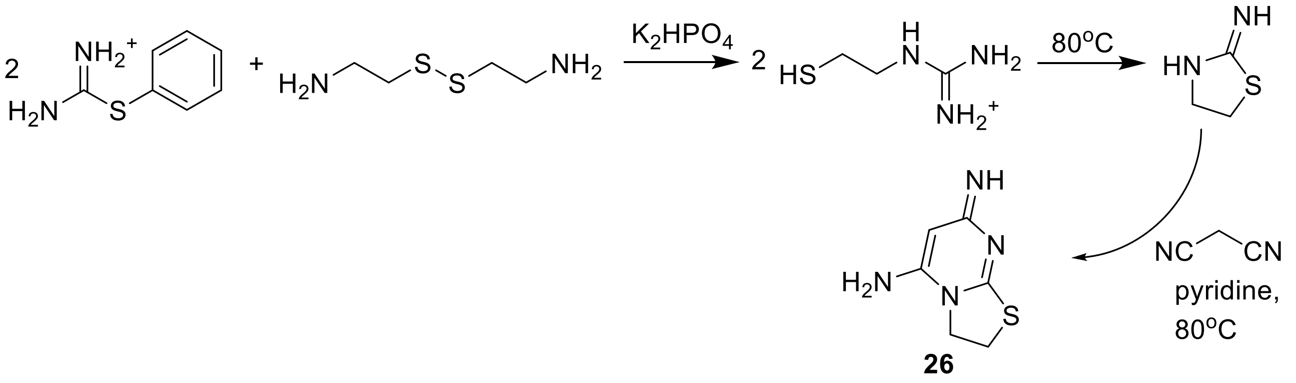

233 mg (1 mmole) of phenylthiouronium bromide was mixed with 117 mg (0.5 mmole) of cystamine dihydrochloride and dissolved in 5 mL of 2M K_2HPO_4. Reaction was left to stand for 2 hours to ensure the completion of autocatalytic ligation. Then the temperature was raised to 80°C and the reaction was kept overnight. Formation of thiazolidine-2-imin was confirmed with ^{1}H NMR. Then 300 mg of malonitrile (4.5 mmole), in 3 mL of pyridine, was added to the reaction. The reaction was kept under vigorous stirring for another 6 hours. Then pyridine and most of the water were evaporated. The products were extracted with 10 mL of EtOH. After evaporation part of the residue was dissolved in DMSO and ^{1}H NMR was recorded.

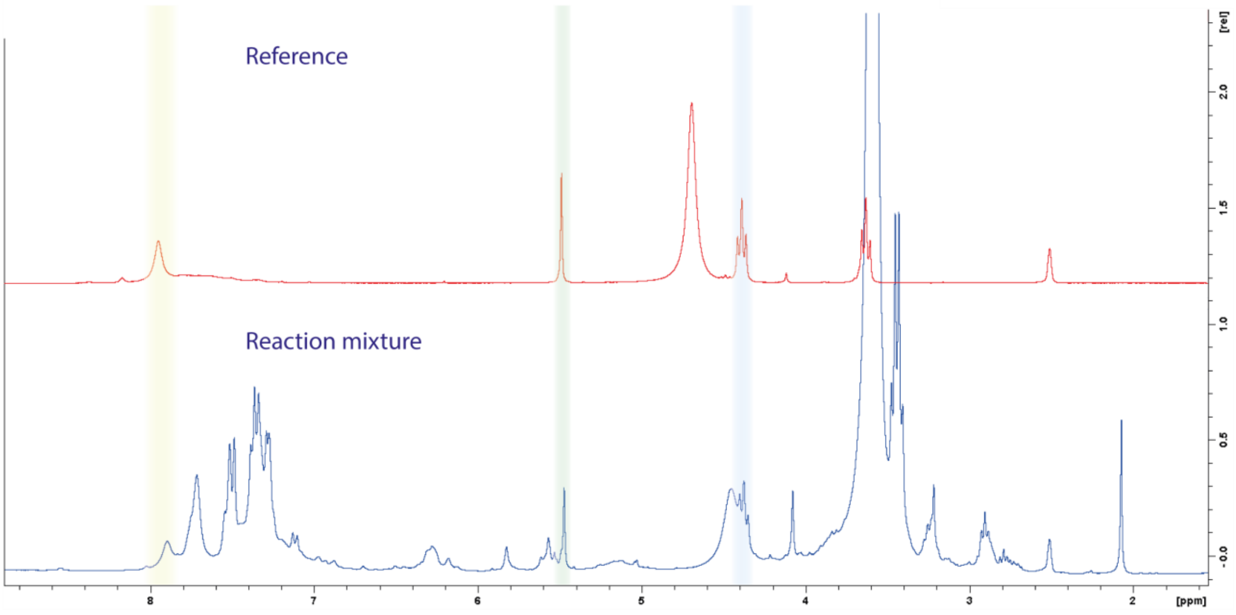

Supplementary Figure 27. Comparison of 1H NMR spectra from the reaction mixture of one-pot synthesis (lower) of **26** to its reference spectrum (upper). Highlighted regions display the match of the reaction mixture with the reference spectrum of the target compound.

6. Experiments in flow

6.1. Flow set-up

The flow set-up consisted of four main components: (i) syringe pumps, (ii) a micro continuously stirred tank reactor (CSTR), (iii) a microfluidic mixer (or two mixers), and (iv) a flow cell. We used NEMESYS Low Pressure Syringe pumps from CETONI in all our experiments. The construction of the micro-CSTR, microfluidic mixers and the flow cell is described in detail below.

Micro-CSTR. The reactor consisted of the main glass body, a glass bottom, rubber connectors to tubing, and a stirring bar. The glass body was manufactured by Witeg Co. with a custom design. The bottom consists of ~0.2 mm cover glass that was glued to the main body with silicon glue after the stirring bar (4/1 mm) had been inserted into the main body. The tubing connectors were made from rubber plungers from 1 ml plastic syringes by punching holes for tubing in the middle of them. To control the temperature inside the reactor, it was installed on a copper plate that had a water circulation system connected to a thermostat (Fig. 28). The copper plate was equipped with a clamping system and thermal paste (CPU grease) was used for better heat transfer. Importantly, the CSTR outlet tubing was made of polyethylene with an inner diameter of 0.4 mm to reduce the transfer time from CSTR to the mixer.

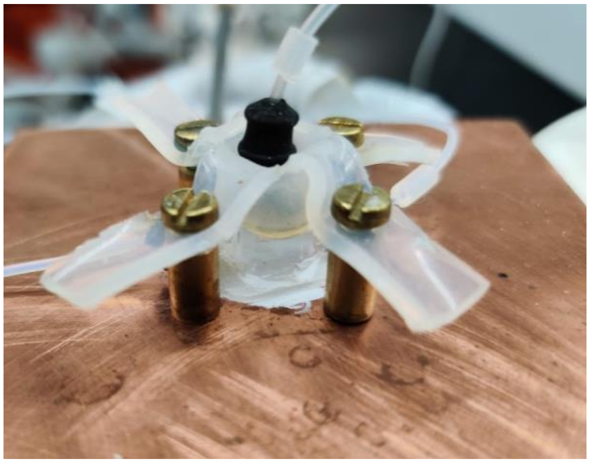

Supplementary Figure 28. Continuous stirred-tank reactor installed on the thermostated copper plate.

Microfluidic mixers. We made two types of microfluidic mixers: (i) with two inlets (required for all the experiments); (ii) with three inlets (required only for experiments studying the response to chemical stimuli). The fabrication protocol for the device with three inlets is the same and it will be described for the device with two inlets. First, three hypodermic needles were cut to ~1 cm length. The needles were placed in a 5 cm plastic

petri dish in such a way that the ends of two of them touch each other and the end of the third needle is ~1 cm from the junction. Next, the petri dish was filled with a mixture of Sylgard® 184 silicone elastomer with 9 wt% of the curing agent. The mixture was degassed under reduced pressure and cured for 2 hours at 65 °C to form a polydimethylsiloxane (PDMS) elastomer. After curing, the needles were removed and PDMS was removed from the petri dish. The channel between the junction and the third inlet was manually scratched, and the device was plasma bonded to a microscope glass slide (Fig. 29).

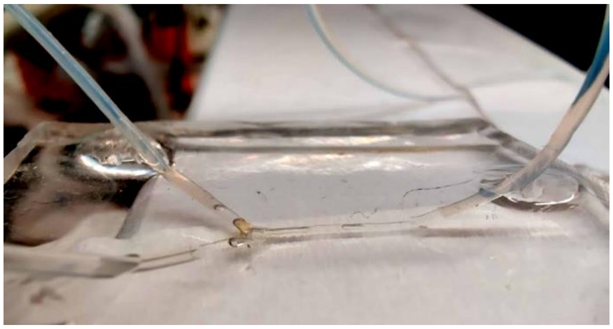

Supplementary Figure 29. Static mixer made of PDMS.

Flow cell. Initially we used a commercially available Helma flow cell with a 0.2 mm light pass; however, it produced unsatisfactory results, most likely because of the non-homogeneous flow in the channel with a high aspect ratio (0.2/4 mm) and at very low flow rates, which characterizes our experiments. In addition, rectangular cells (similar to the ones we used previously)[11] are prone to the accumulation of gas bubbles. Therefore, we decided to build a flow cell with a round cross-section, which would provide most of the homogeneous flow, despite its ability to refract light. Since we analyzed the changes in the concentration of the same compound (2-nitro-5-thiobenzoate) in the same solution (phosphate buffer), the problems associated with calculating the absorbance can be partially resolved by using a nonlinear calibration curve. To build the cell, we used a glass capillary with a 1 mm internal diameter. Using a gas burner, we made two necks 1 cm apart in this capillary. The capillary was cut at these necks, and two needles (d = 0.8 mm) were inserted into the necks. The connections of needles to the capillary were sealed with a minimal amount of silicone hermetic. The capillary, together with needles inserted into it, was placed into a petri dish on top of two metal cylinders (1.2 mm in diameter, made from thick needles). The PDMS elastomer was poured on top of the whole assembly and cured. Once PDMS was cured, the rectangular piece of PDMS was cut and the needles were removed, leaving a connection for the tubing (Fig. 30). Finally, the cell was attached to a glass slide and placed in the focus of the beam of the UV-Vis spectrometer.

We noted that a more convenient way of embedding a glass capillary into PDMS would be to use a two-stage process that eliminates the use of metal cylinders as support for the capillary. Here, one needs to make a cured layer of PDMS in a petri dish and to place the capillary directly on top of it.

The embedding of the capillary into PDMS plays a critical role for this cell; it eliminates sharp changes in the refractive index at the boundary of the round capillary because the refractive index of glass (~1.52) is much closer to the refractive index of PDMS (1.42) than to the refractive index of air (1). Therefore, it allows minimizing the refraction of the beam to an acceptable level.

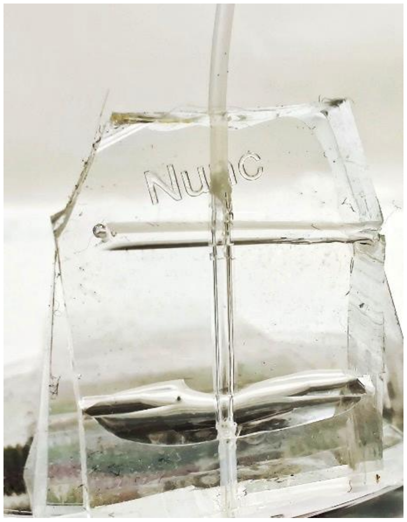

Supplementary Figure 30. Handmade flow cell for UV-vis made of glass capillary in a piece of PDMS polymer.

Calibration of the flow cell. We used the calibration curves to convert the absorption to the concentration values and thus, to quantify the UV data from the oscillatory reactions in CSTR (Fig. 31). To obtain the calibration curves, we mixed one volume of the mercaptoethanol solution of a corresponding concentration in phosphate buffer pH 8 with three volumes of the water/methanol (1.5/1) solution of Ellman's reagent (16.6 mM) also containing KH_2PO_4 (146 mM). Then, we set the absorbance to 0 for pure water, passed the resulting solution through our self-made flow cell, and recorded the absorbance of the solutions. The whole sequence of preparing the solution and measuring the absorbance was repeated three times for each concentration of mercaptoethanol. The mean values were plotted and fitted with the following function:

$$A = a - b \cdot d^{[C_{thiols}]},$$

where A is the absorbance, C_{thiols} is the concentration of thiols, and a, b, d are the parameters.

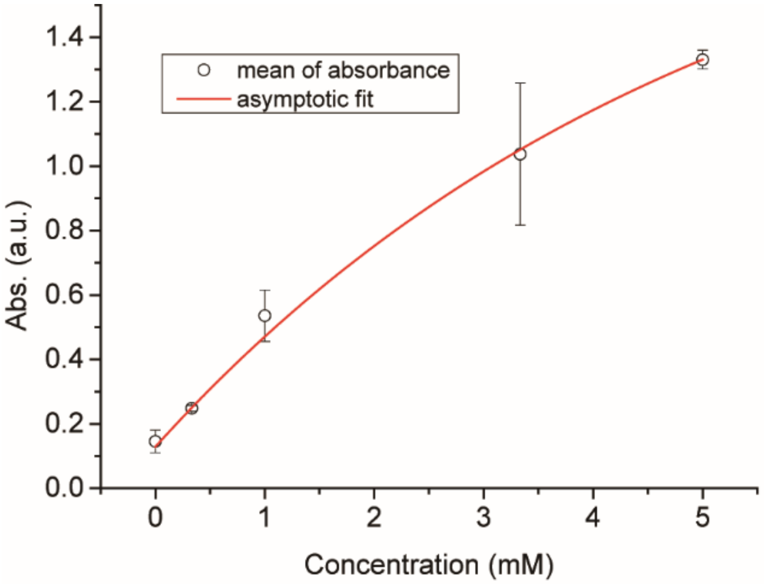

Supplementary Figure 31. Calibration curves of thiol concentration in a home-made flow cell using Ellman's reagents. The background was corrected by substracting the absorbance of phosphate buffer. Nonlinear fitting of the asymptotic function ($y = a - b*c^x$) was performed using *Origin 9*. The fitted parameters are as follows: (a) a = 2.06 ± 0.45, b = 1.93 ± 0.43, c = 0.82 ± 0.06. The error bars represent the standard deviations.

6.2. The standard protocol for oscillations in flow (except for experiments that study the response to chemical stimuli)

Four syringes were filled with the required solutions as described below:

- 163 mg of thiouronium salt **8** were dissolved in 2.5 ml of HPLC-grade water, and this solution was transferred into a syringe. An additional 0.5 ml of water was used to wash the vial in which the solution was prepared, and the volume in the solution in the syringe was precisely filled to 3 ml. The same method was used to control the total volume of the solutions when filling the other syringes. The final concentration of thiouronium salt **8** was 168 mM (in syringe) in all experiments.
- 205 mg of acrylamide and maleimide or $K_3[Fe(CN)_6]$ in various amounts were dissolved in water. The solution was transferred to a syringe and its volume was adjusted to 3 ml. Final concentrations in the syringe were 962 mM for acrylamide and 12 mM, 18.6mM, 24mM, and 30mM for maleimide or 16.6 mM for $K_3[Fe(CN)_6]$.
- Phosphate buffer (3M) was used to prepare the solution for the third syringe. The buffer was prepared to have pH 8 after a threefold dilution to a concentration of 1 M. Thus, to obtain 100 ml of a buffer solution, 4.325 g (0.0318 mol) of anhydrous KH_2PO_4 and 46.347 g (0.2679 mol) of anhydrous K_2HPO_4

were placed in a 100 ml volumetric flask and filled with HPLC grade water up to 100 ml. This PBS was used to prepare a 3 ml solution of 186 mg of cystamine dihydrochloride salt in a third syringe. The final concentration of cystamine in the syringe was 276 mM for all experiments.

- For filling the last syringe, two solutions were prepared separately. First, 10 ml of HPLC grade methanol was used to dissolve 164 mg (414 mmole) of 5,5'-dithiobis-(2-nitrobenzoic acid) (Ellman's reagent) and 15 ml of HPLC grade water was used to dissolve 0.5 g of KH_2PO_4. Those two solutions were combined and a 25 ml glass syringe was filled with solution through a 0.22 µm syringe filter. The final concentration of Ellman's reagent in the syringe was 16.6 mM.

All 5 ml syringes (with 3 ml of solution each) were installed in the syringe pump system and connected to the CSTR using 0.5 mm internal diameter PTFE tubing. The CSTR outlet tubing was connected to the microfluidic mixer where the content of CSTR was mixed with Ellman's reagent. In all experiments, the flow of the solution of Ellman's reagent was three times higher than the flow from CSTR. After mixing, the flow passed through the flow cell, where absorbance at 412 nm was measured.

Experiments with the oscillator based on the disulfide **18** were performed in the same way, but the composition of syringes was changed because of the phase separation of the deprotonated **18** in 3M phosphate buffer. Thus, the content of the syringes was as following: syringe 1 (**18** in form of dihydrochloride, 168 mM); syringe 2 (phosphate buffer, K+, 3M, pH 8); syringe 3 (**8** 168 mM, maleimide 24 mM, acrylamide 336 mM). No modifications were made to the syringe with Ellman's reagent and the detection system. To analyze oscillation is bicycle **22**, an individual drops (~ 15 µL each) of solution were collected directly from the outlet of CSTR, diluted with 50 µL of water containing 1 mM of BocLysine as internal standard (although we found during analysis that stability of the MS detector was excellent and the use of the standard was not necessary), and analyzed by HPLC-MS (SILEC Primeser 500 4.6x250 mm, H_2O(0.1 % TFA)/CH_3CN). The peak at 4.44 min corresponded to bicycle **22** as we confirmed by comparing it with the peak in the chromatogram of the standard obtained by cyclization of the 3-(2-iminothiazolidin-3-yl)propanenitrile.

6.3. Protocol for studies of responses of oscillators to chemical stimuli

To study the response of oscillators to chemical stimuli, we developed a system that allowed us to add an extra reagent to the oscillatory mixture while keeping the concentrations of all other components constant. In this system, the content of the syringes that supply cystamine to the phosphate buffer, and Ellman's reagent remained the same as described in the previous section. $K_3[Fe(CN)_6]$ (5 mM) was added to the syringe containing maleimide (5 mM) and acrylamide (321 mM) for the experiment with a "mixed" oscillator. These three syringes were connected to the CSTR and the mixer identically to the original set-up. However, the inlet of the CSTR through which **8** was supplied in the original set-up was connected to the outlet of a mixer with

three inlets (see Figure 6 in the paper). These three inlets were connected to syringes with (i) **8** (336 mM) or a mixture of **8** (150 mM) with acetylthiocholine (150 mM) in water, (ii) pure water, (iii) a solution of the compound; thus, it was the effect we intended to study (i.e., furfuryl alcohol (600 mM), thiosemicarbazide (120 mM), glyoxal (300 mM)). The flow from the syringe (i) was always ½ from the flow from syringes connected directly to CSTR. The combined flow from syringes (ii) and (iii) was also ½ from the flow from syringes connected directly to CSTR. By changing the ratio of flows from syringes (ii) and (iii), we controlled the dilution and consequently, the final concentrations of the CSTR in the furfuryl alcohol, thiosemicarbazide, and glyoxal.

6.4. Data processing for the oscillatory experiments

The results of the oscillatory experiments were then plotted using *MATLAB 2019a*; they are presented in the following Supplementary Figures 32 to 35. The script "plotall.m" has the following operations: (1) it loads the data files and removes noise from the original data, (2) it checks the baselines and uses the corresponding methods to convert the absorbance to the concentration, and (3) it saves the converted files as new data files with the names "nonoiseCon" plus the original name. This script also displays the data in time-concentration plots just for debugging purposes. The other *MATLAB* file, "plotOscillator.m", plots each of the oscillation experimental results, which corresponded to the data points in the phase plot in Figure 6d in the paper.

6.5. Plots of oscillations for all experiments.

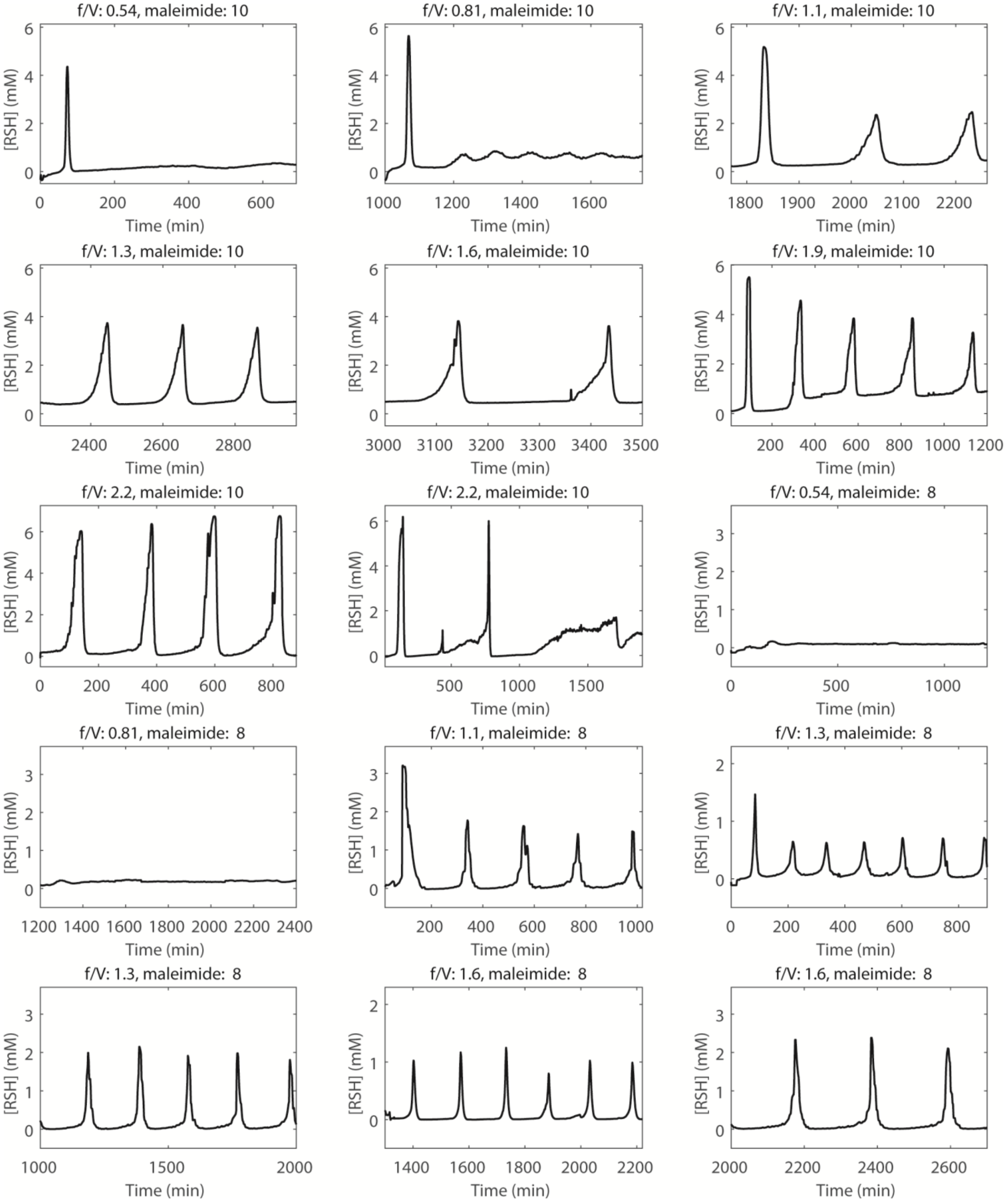

Supplementary Figure 32. Summary of the oscillatory experiments shown in Figure 6d. Reaction conditions: 1 M phosphate buffer pH 8, [**8**] = 56 mM,[CSSC] = 92 mM, [acrylamide] = 321 mM. The units of space velocity (f/V) and the maleimide concentration are $10^{-4}\times S^{-1}$ and mM, respectively. The different behaviors of the system at different concentrations (from 10 mM to 4 mM) of maleimide and different space volocities (f/v, from $0.54\times10^{-4}\times S^{-1}$ to $3.8\times10^{-4}\times S^{-1}$) are shown.

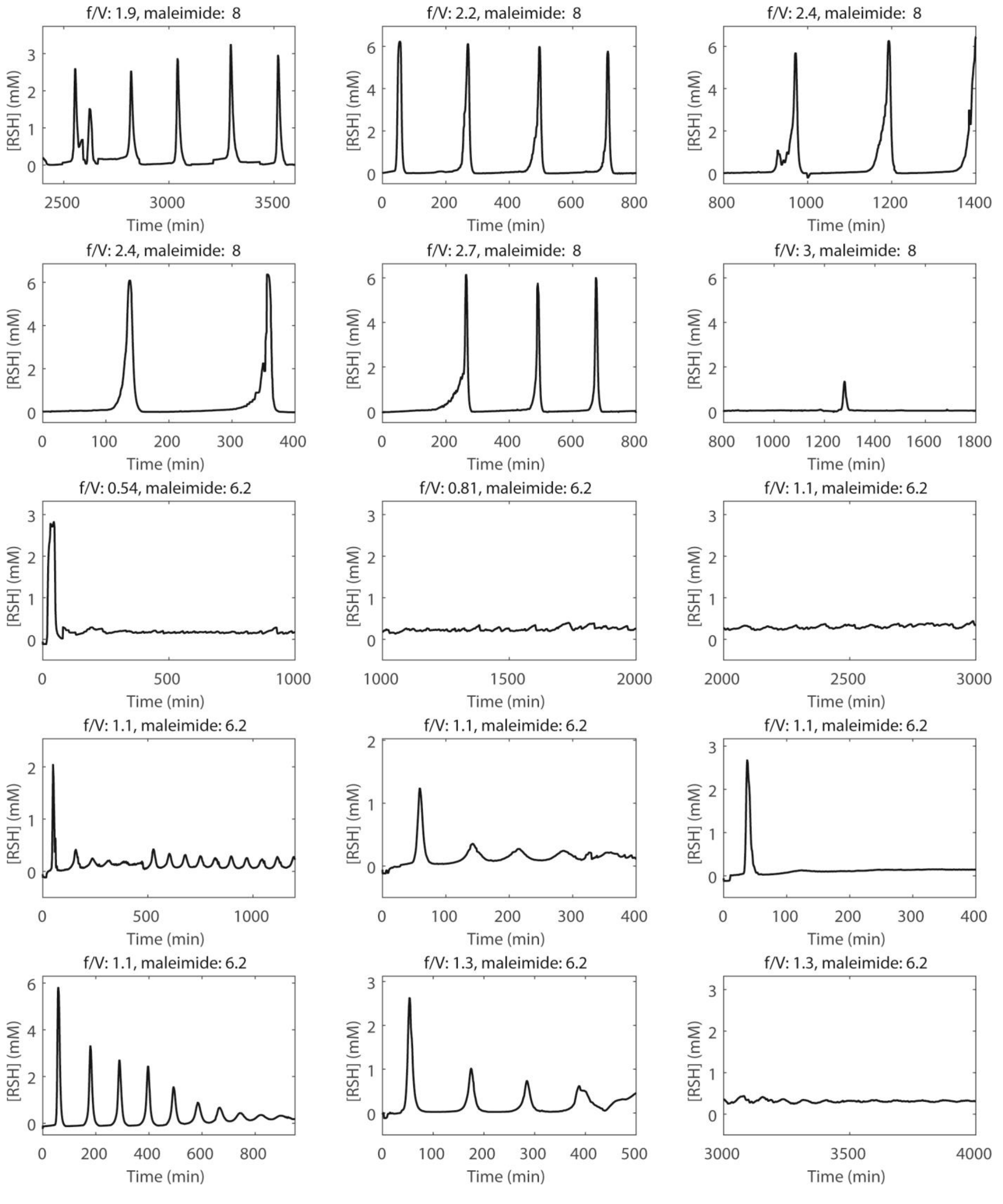

Supplementary Figure 33. Continuation of Fig. 32.

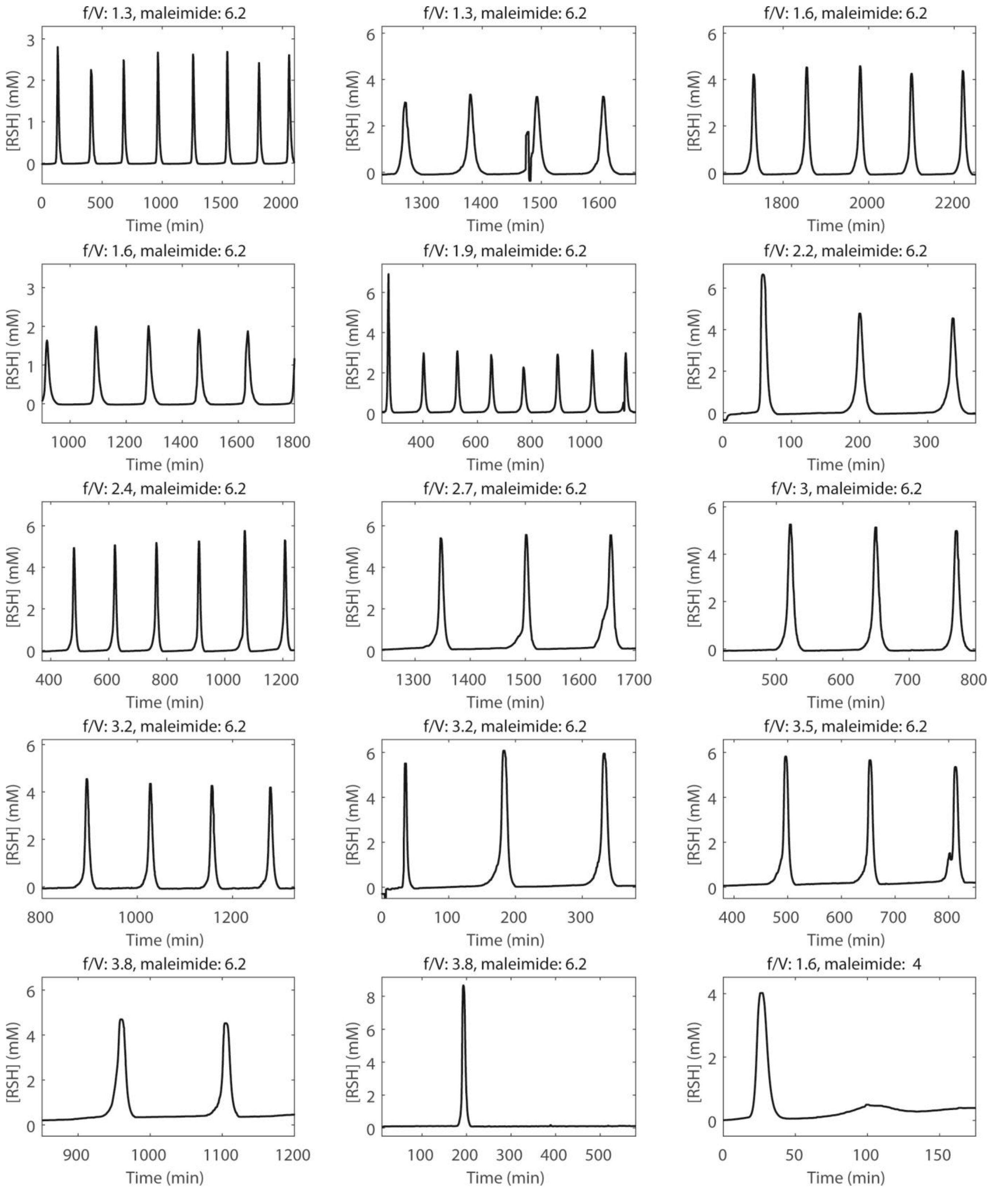

Supplementary Figure 34. Continuation of Fig. 33.

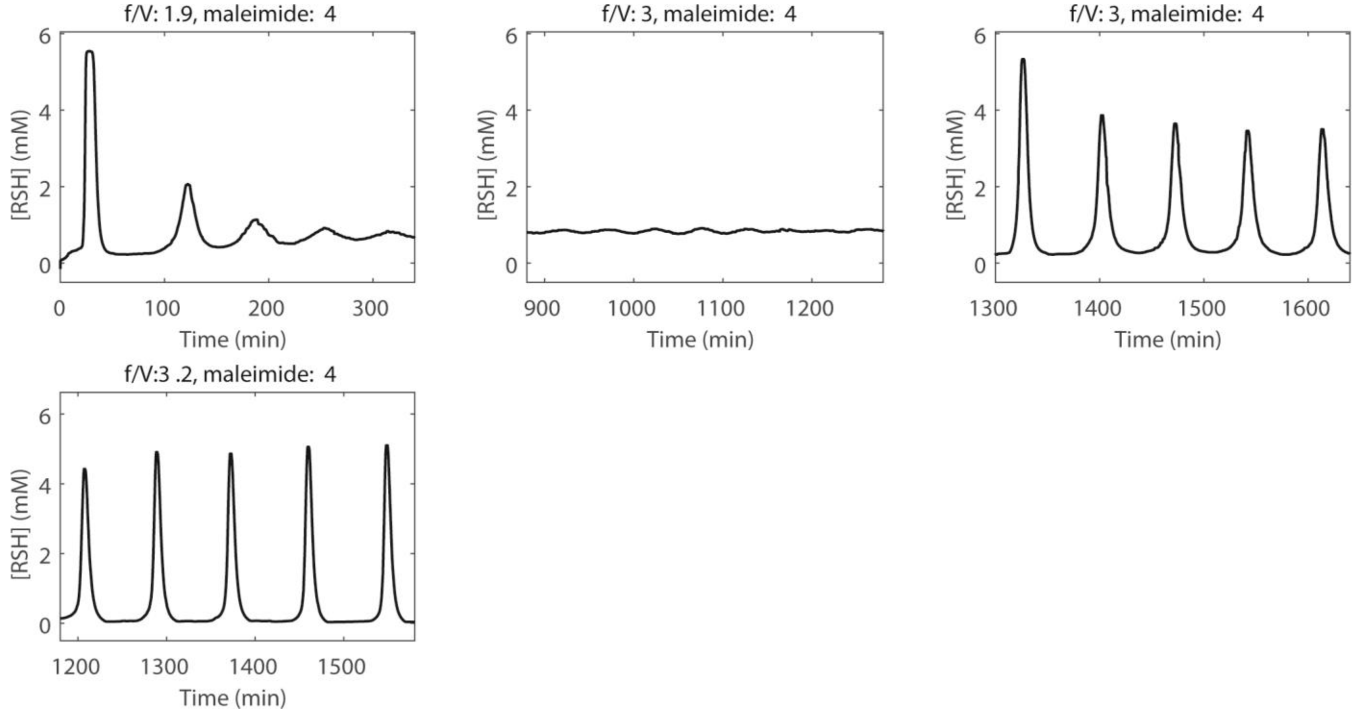

Supplementary Figure 35. Continuation of Fig. 34.

7. Modeling

7.1. Model of the autocatalytic reactions

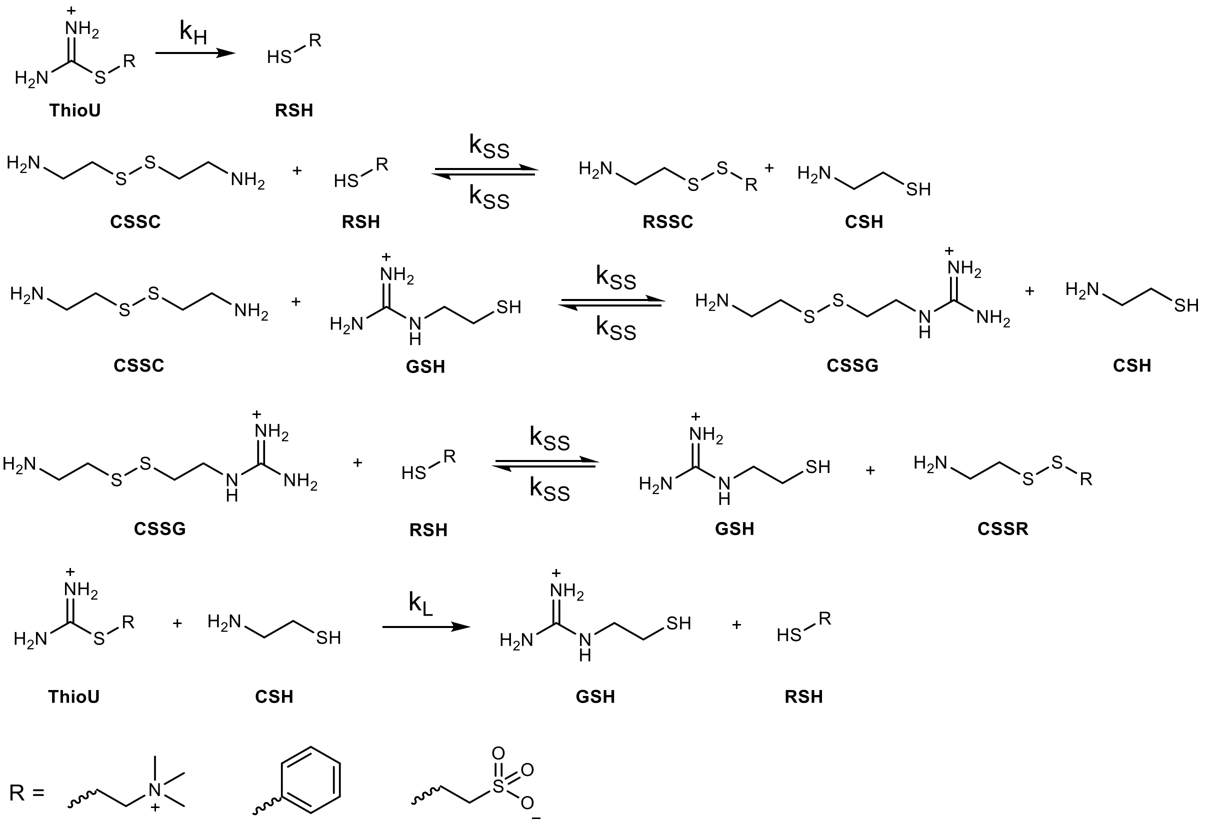

Supplementary Figure 36. Details of the numerical model for the autocatalytic reaction between thiouronium salts and cystamine (**17**). Three parameters are involved: k_H is the rate of the thiouronium salt hydrolysis; k_L is the rate of the ligation; k_{SS} is the rate of the disulfide exchange. All the reactions in the model are assumed to obey the mass-action law.

The numerical model of the reaction between thiouronium salts and **17** is shown in Fig. 36. Here, three assumptions were made: First, all disulfide exchanges have the same rate constants. Second, the six disulfides can be reduced to three, which have a common part of $[CSH]$. Therefore, the disulfide exchanges are also simplified to three exchange reactions that involve $[CSH]$-disulfides. Accordingly, the concentration of cystamine (it contains two $[CSH]$ parts) in the model was twofold higher than the experimental value. Third, two stages of the thiol-assisted amination of thiouronium salts were not separated in this model; instead, they were treated as one irreversible reaction. The dynamics of the model is described by the following system of ordinary differential equations (ODEs) (5) – (11):

$$\frac{d[ThioU]}{dt} = -k_H[ThioU] - k_L[ThioU][CSH] \quad (5)$$

$$\frac{d[CSSC]}{dt} = -k_{SS}[CSSC][RSH] - k_{SS}[CSSC][GSH] + k_{SS}[RSSC][CSH] + k_{SS}[CSSG][CSH] \quad (6)$$

$$\frac{d[RSH]}{dt} = k_H[ThioU] - k_{SS}[CSSC][RSH] - k_{SS}[CSSG][RSH] + k_L[ThioU][CSH] + k_{SS}[RSSC][CSH] + k_{SS}[CSSR][GSH] \quad (7)$$

$$\frac{d[CSH]}{dt} = -k_{SS}[RSSC][CSH] - k_{SS}[CSSG][CSH] - k_L[ThioU][CSH] + k_{SS}[CSSC][GSH] + k_{SS}[CSSC][RSH] \quad (8)$$

$$\frac{d[RSSC]}{dt} = -k_{SS}[RSSC][CSH] + k_{SS}[CSSG][RSH] - k_{SS}[CSSR][GSH] + k_{SS}[CSSG][RSH] (9)$$

$$\frac{d[GSH]}{dt} = -k_{SS}[CSSC][GSH] + k_{SS}[CSSG][CSH] + k_L[ThioU][CSH] - k_{SS}[CSSR][GSH] + k_{SS}[CSSG][RSH] \quad (10)$$

$$\frac{d[CSSG]}{dt} = -k_{SS}[CSSG][CSH] + k_{SS}[CSSC][GSH] - k_{SS}[CSSG][RSH] + k_{SS}[CSSR][GSH] \quad (11),$$

where k_H is the rate of thiouronium salt hydrolysis, k_{SS} is the rate of the disulfide exchange, and k_L is the overall rate of the ligation. The model was built in COPASI and parameters were fitted using the build-in function of the parameter estimation. Here, k_{SS} was fixed to 0.444 ($M^{-1}S^{-1}$) according to our previous research.[11] The other two parameters (k_H and k_L) were estimated from least squares curve fitting and the Levenberg–Marquardt algorithm was used. The COPASI model was attached as a separate file.

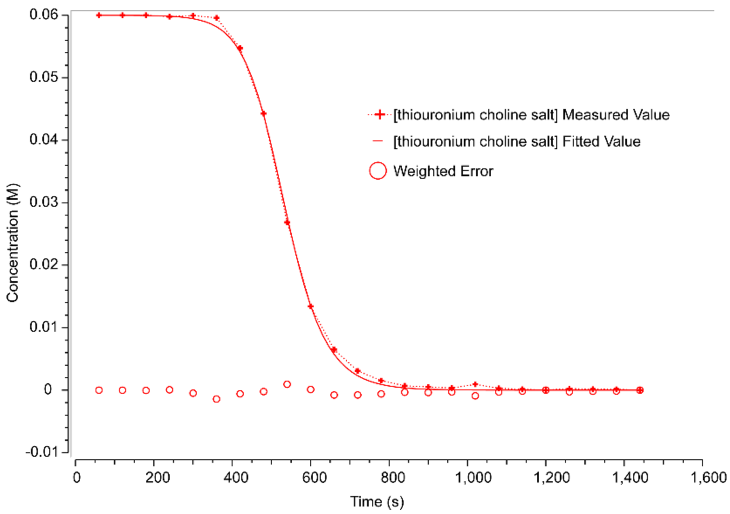

Supplementary Figure 37. Parameter estimation for the reaction between **8** (60 mM) and **17** (60 mM) in phosphate buffer pH 8. k_H = 4.58×10^{-7}±4.71×10^{-8} (s^{-1}) and k_L = 0.78±0.02 (M^{-1}s^{-1}) was obtained after parameter fitting.

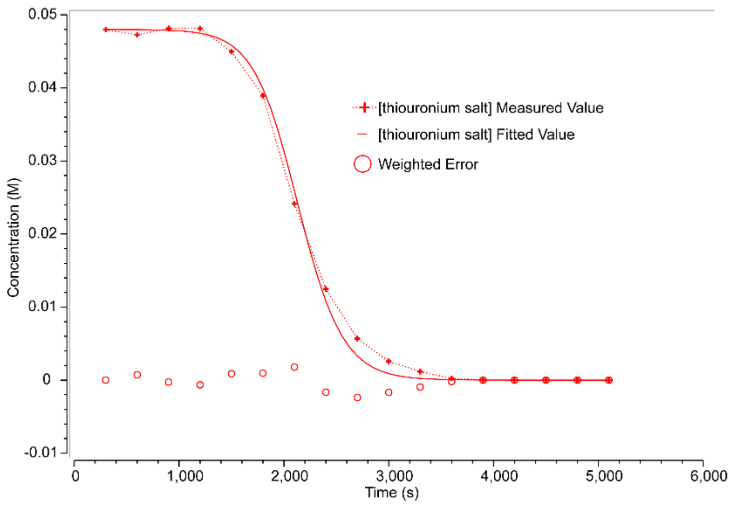

Supplementary Figure 38. Parameter estimation for the reaction between **4** (48 mM) and **17** (47 mM) in phosphate buffer pH 8. k_H = 1.24×10^{-9}±1.16×10^{-9} (s^{-1}) and k_L = 0.23±0.02 (M^{-1}s^{-1}) was obtained after parameter fitting.

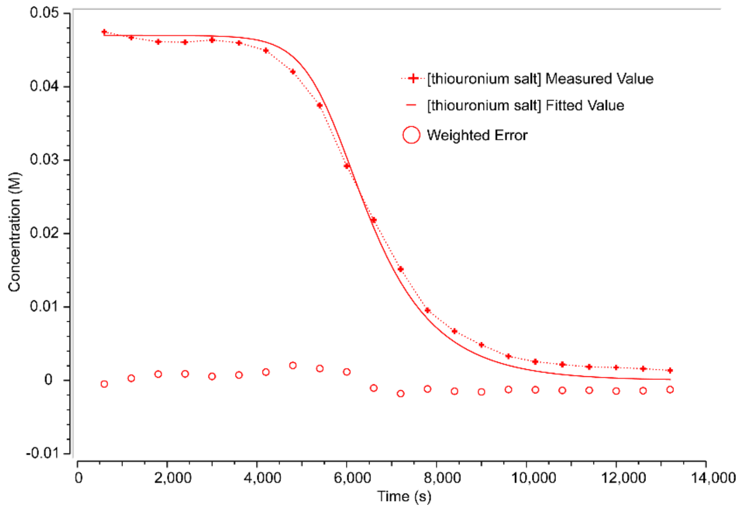

Supplementary Figure 39. Parameter estimation for the reaction between **3** (47 mM) and **17** (48 mM) in phosphate buffer pH 8. k_H = 8.04×10^{-8}±1.23×10^{-8} (s^{-1}) and k_L =0.038±0.00064 (M^{-1}s^{-1}) was obtained after parameter fitting.

7.2. Complete model of the oscillator

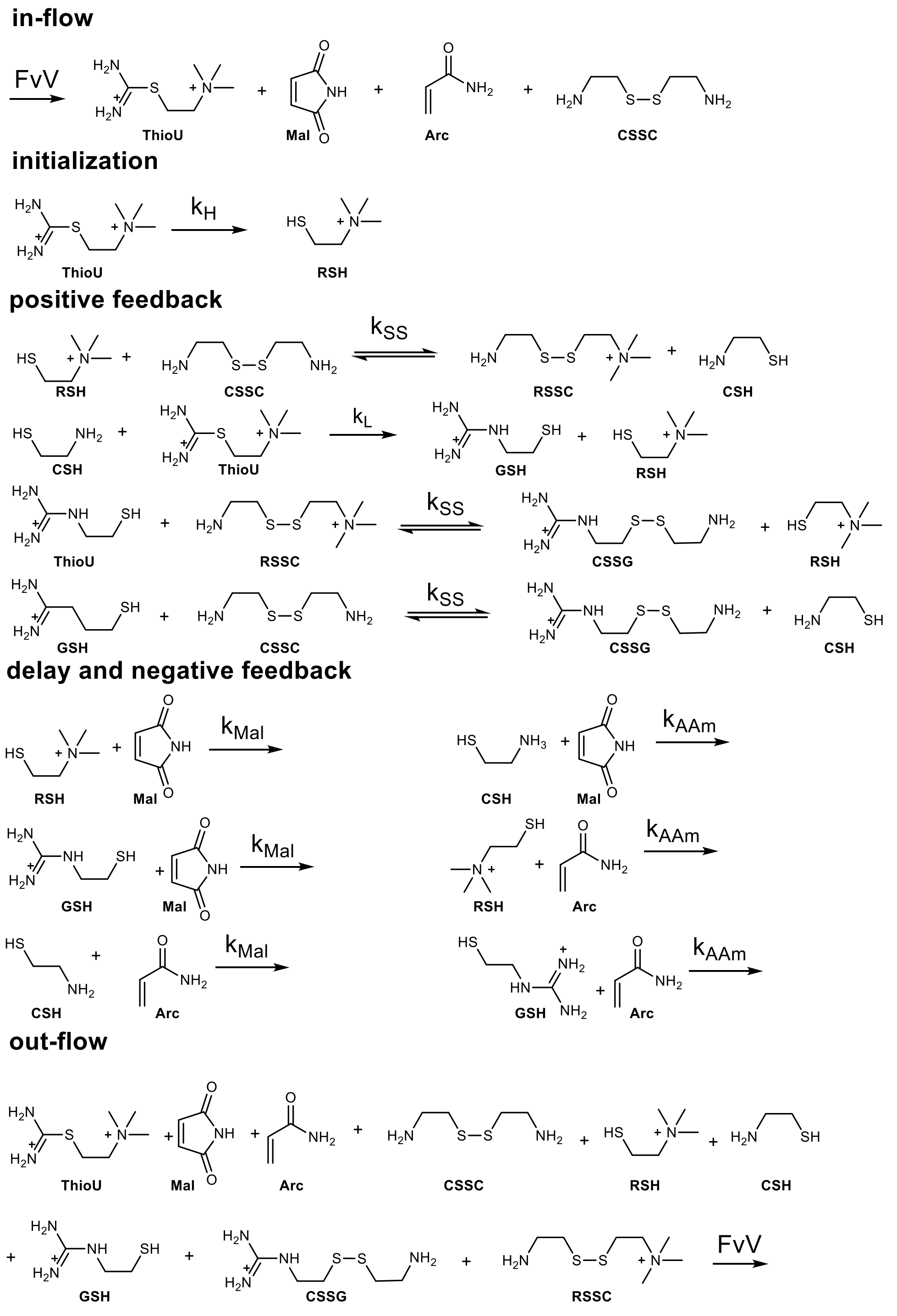

Supplementary Figure 40. The model used for the numerical simulation of the oscillation in CSTR. Three modules were added to the autocatalytic model, the in and out-flow, delay by maleimide, and negative feedback using acrylamide. The parameters involved here are as follows: k_H is the rate of the thiouronium salt hydrolysis; k_L is the rate of the ligation; k_{SS} is the rate of the disulfide exchange; k_{AAm} is the rate of the reaction between thiol and acrylamide; k_{Mal} is the rate of the reaction between thiol and maleimide, and FvV is the space velocity.

All reactions except for flow are assumed to follow the mass-action law. The rate law for flow supply and removal is described as FvV × concentration.

The numerical model of the oscillation in CSTR (Fig. 40) was built according to the previous literature,[11] which was simulated using *MATLAB* (Fig. 41-43). The behavior of the system was described by a set of ODEs and was solved numerically by *MATLAB*'s build-in function ode45. The parameters in Supplementary Figures 41-43 were set as follows: A(inputTU) = 0.05 M (the initial concentration of thiouronium salt in the input stream); A(inputDCys) = 0.1 M (the initial concentration of cystamine in the input stream); A(inputMal) = 0.010 M (the initial concentration of malemide in the input stream); A(inputAAm) = 0.30 M (the initial concentration of acrylamide in the input stream); k_{AAm} = 0.014 ($M^{-1}s^{-1}$); k_{Mal} = 150 ($M^{-1}s^{-1}$); k_{SS} = 0.45 ($M^{-1}s^{-1}$); k_H = 7e^{-5} s^{-1}, FvV = 1.5×10^{-4} s^{-1}. Supplementary Figure 41 shows the sustained oscillation of the concentration of all thiols when using **8** (k_L = 0.78 ($M^{-1}s^{-1}$)). Supplementary Figure 42 describes the damped oscillation of the concentration of all thiols when using **4** (k_L = 0.23 ($M^{-1}s^{-1}$)). Supplementary Figure 43 describes the single oscillation of the concentration of all thiols when using **3** (k_L = 0.038 ($M^{-1}s^{-1}$)).

The dynamics of the model is described by the following system of ordinary differential equations (ODEs) (12) – (20):

$$\frac{d[ThioU]}{dt} = -f_vV[ThioU] - k_H[ThioU] - k_L[ThioU][CSH] + f_vV[ThioU]_0 \quad (12)$$

$$\frac{d[CSSC]}{dt} = -f_vV[CSSC] - k_{SS}[CSSC][RSH] - k_{SS}[CSSC][GSH] + k_{SS}[RSSC][CSH] + k_{SS}[CSSG][CSH] + f_vV[CSSC]_0 \quad (13)$$

$$\frac{d[RSH]}{dt} = -f_vV[RSH] - k_{Mal}[Mal][RSH] - k_{AAm}[Arc][RSH] + k_H[ThioU] - k_{SS}[CSSC][RSH] - k_{SS}[CSSG][RSH] + k_L[ThioU][CSH] + k_{SS}[RSSC][CSH] + k_{SS}[CSSR][GSH] \quad (14)$$

$$\frac{d[CSH]}{dt} = -f_vV[CSH] - k_{Mal}[Mal][CSH] - k_{AAm}[Arc][CSH] - k_{SS}[RSSC][CSH] - k_{SS}[CSSG][CSH] - k_L[ThioU][CSH] + k_{SS}[CSSC][GSH] + k_{SS}[CSSC][RSH] \quad (15)$$

$$\frac{d[RSSC]}{dt} = -f_vV[RSSC] - k_{SS}[RSSC][CSH] + k_{SS}[CSSG][RSH] - k_{SS}[CSSR][GSH] + k_{SS}[CSSG][RSH] \quad (16)$$

$$\frac{d[GSH]}{dt} = -f_vV[GSH] - k_{Mal}[Mal][GSH] - k_{AAm}[Arc][GSH] - k_{SS}[CSSC][GSH] + k_{SS}[CSSG][CSH] + k_L[ThioU][CSH] - k_{SS}[CSSR][GSH] + k_{SS}[CSSG][RSH] \quad (17)$$

$$\frac{d[CSSG]}{dt} = -f_v V[CSSG] - k_{SS}[CSSG][CSH] + k_{SS}[CSSC][GSH] - k_{SS}[CSSG][RSH] + k_{SS}[CSSR][GSH] \quad (18)$$

$$\frac{d[Mal]}{dt} = -f_v V[Mal] - k_{Mal}[Mal][GSH] - k_{Mal}[Mal][CSH] - k_{Mal}[Mal][RSH] + f_v V[Mal]_0 \quad (19)$$

$$\frac{d[Arc]}{dt} = -f_v V[Arc] - k_{Mal}[Arc][GSH] - k_{Mal}[Arc][CSH] - k_{Mal}[Arc][RSH] + f_v V[Arc]_0 \quad (20),$$

where k_{Mal} is the rate of the reaction between maleimide and free thiol, k_{AAm} represents the rate of the reaction between acrylamide and free thiol, and $f_v V$ is the space velocity of CSTR. $[Mal]_0$, $[Arc]_0$, $[CSSC]_0$, and $[ThioU]_0$ are the flow-in concentrations of maleimide, acrylamide, **17**, and thiouronium salts, respectively.

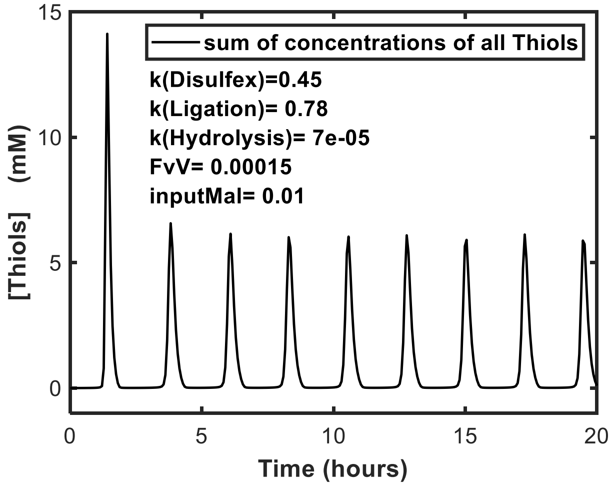

Supplementary Figure 41. Numerical simulation of the oscillation in CSTR when applying **8**.

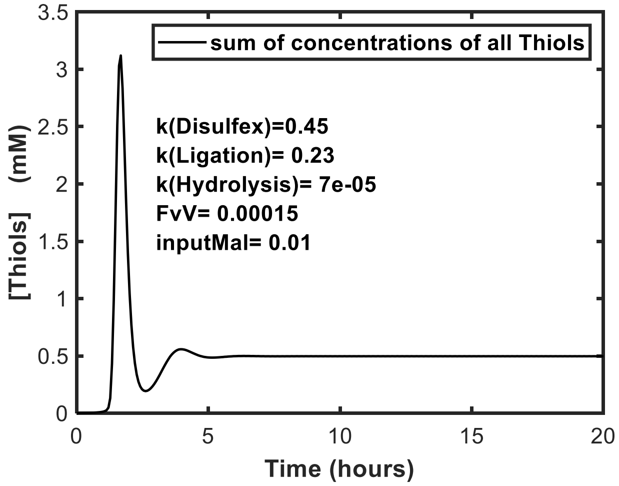

Supplementary Figure 42. Numerical simulation of the oscillation in CSTR when applying **4**.

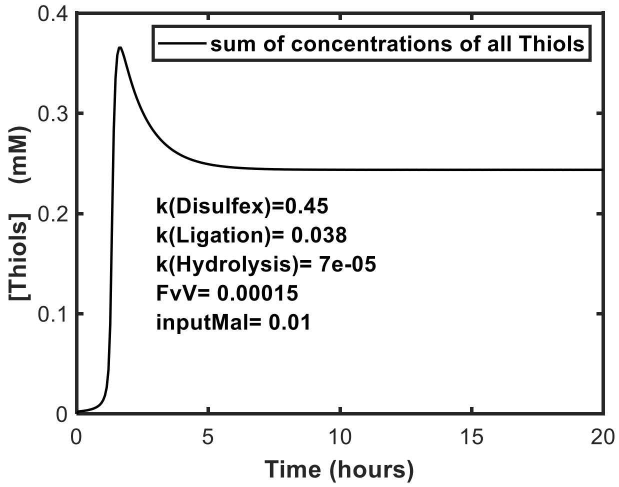

Supplementary Figure 43. Numerical simulation of the oscillation in CSTR when applying **3**.

7.3. Three-variable model and linear stability analysis of the oscillator

To analyze the behavior of the oscillator, we used the three-variable model, which is identical to the one used in the analysis of the thioester-based oscillator.[11]

The model of the thiouronium salt oscillator is reduced to three variables by applying three approximations: first, the positive feedback loop is described by simple quadratic autocatalysis with the rate constant k_1; second, the negative feedback loop (the reaction with acrylamide) is described by a first-order reaction with rate constant k_3, and third, the end products can be neglected.

With these assumptions, the system of reactions can be described by:

$$
\begin{aligned}
S + A &\xrightarrow{k_1} 2A \\
S &\xrightarrow{k_4} A \\
I + A &\xrightarrow{k_2} \varnothing \\
A &\xrightarrow{k_3} \varnothing \\
A &\xrightarrow{k_0} \varnothing \\
I &\xrightarrow{k_0} \varnothing \\
S &\xrightarrow{k_0} \varnothing \\
&\xrightarrow{k_0 I_0} I \\
&\xrightarrow{k_0 S_0} S
\end{aligned}
$$

where A represents $[(Me)_3N^+CH_2CH_2SH]$ + $[(NH_2)_2^+CNHCH_2CH_2SH]$ + [CSH], I represents [Maleimide], S represents $[(NH_2)_2^+CSCH_2CH_2N^+(Me)_3]$, $k_{1\text{-}4}$ are rate constants, and k_0 is the ratio of the flow in the reactor to the volume of the reactor; $\varnothing$ indicates washout or the formation of inactive products. The system of kinetic equations takes the following form:

$$dA/dt = k_1SA - k_2IA - k_3A - k_0A + k_4S \quad (28)$$

$$dI/dt = k_0I_0 - k_0I - k_2IA \quad (29)$$

$$dS/dt = k_0S_0 - k_0S - k_4S - k_1SA \quad (30)$$

The steady states are defined by:

$$\frac{k_1k_0S_0A}{k_0 + k_4 + k_1A} - \frac{k_2k_0I_0A}{k_0 + k_2A} - (k_0 + k_3)A + \frac{k_4k_0S_0}{k_0 + k_4 + k_1A} = 0 \quad (31)$$

To analyze the stability of the steady states, we used the Jacobian matrix (**J**), and determined the eigenvalues (λ) such that the following is not invertible:

$$\begin{matrix} k_1S - k_2I - k_3 - k_0 - \lambda & -k_2A & k_1A + k_4 \\ -k_2I & -k_0 - k_2A - \lambda & 0 \\ -k_1S & 0 & -k_1A - k_0 - k_4 - \lambda \end{matrix}$$

We used a MatLab script (available on request) modified from our previous work to compute and plot a 2D map of the oscillatory and steady-state regions (Fig. 6d in the paper).[12] We noted that the script uses the input parameters "param.K_AAm" and [Acrylamide], which are related to k_3 by k_3 = param.K_AAm*[Acrylamide]. The parameters ($k_1 = 0.507$ $s^{-1}M^{-1}$, $k_2 = 300$ $s^{-1}M^{-1}$, $k_3 = 0.0099$ s^{-1}, $k_4 = 3.7{\cdot}10^{-5}$ s^{-1}.), which we used to construct the 2D map, were obtained by manual optimization of the parameters ($k_1 = 0.507$ $s^{-1}M^{-1}$, $k_2 = 300$ $s^{-1}M^{-1}$, $k_3 = 0.00932$ s^{-1}, $k_4 = 7.09{\cdot}10^{-5}$ s^{-1}.) obtained from the fitting of a single oscillation in the batch experiment (see the previous section).

8. NMR spectra

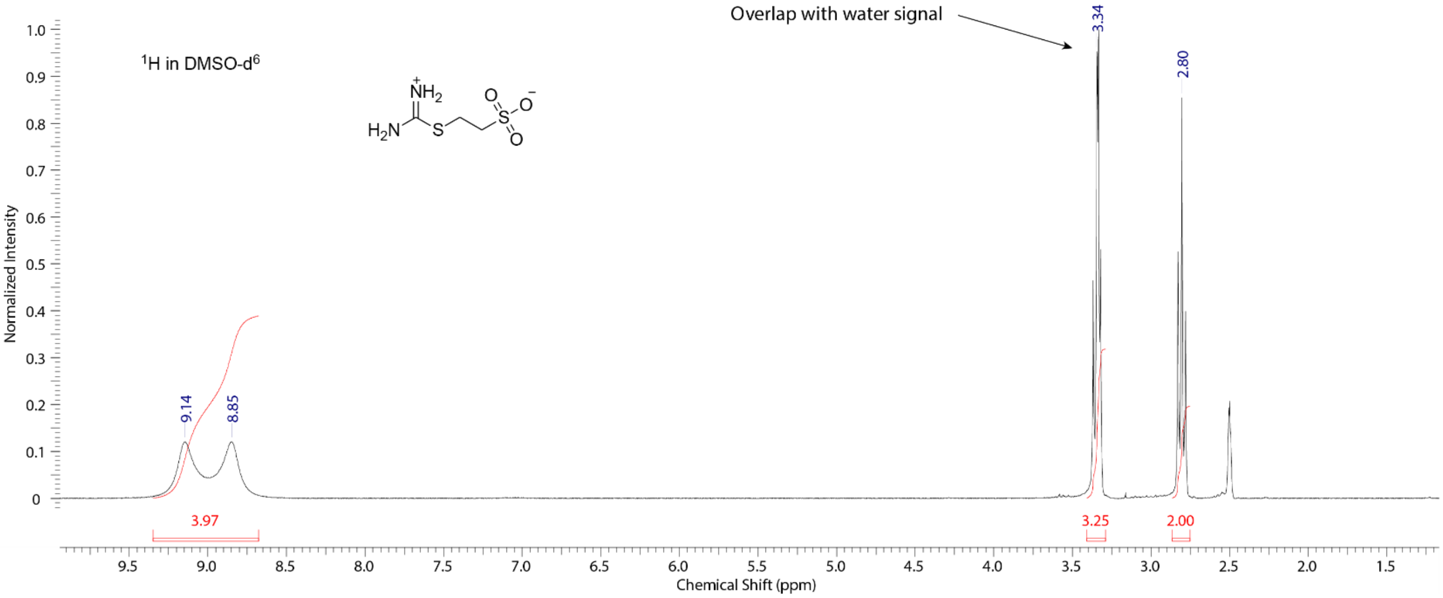

Supplementary Figure 44. ^{1}H NMR (300 MHz, DMSO-d^{6}) of the compound **3**.

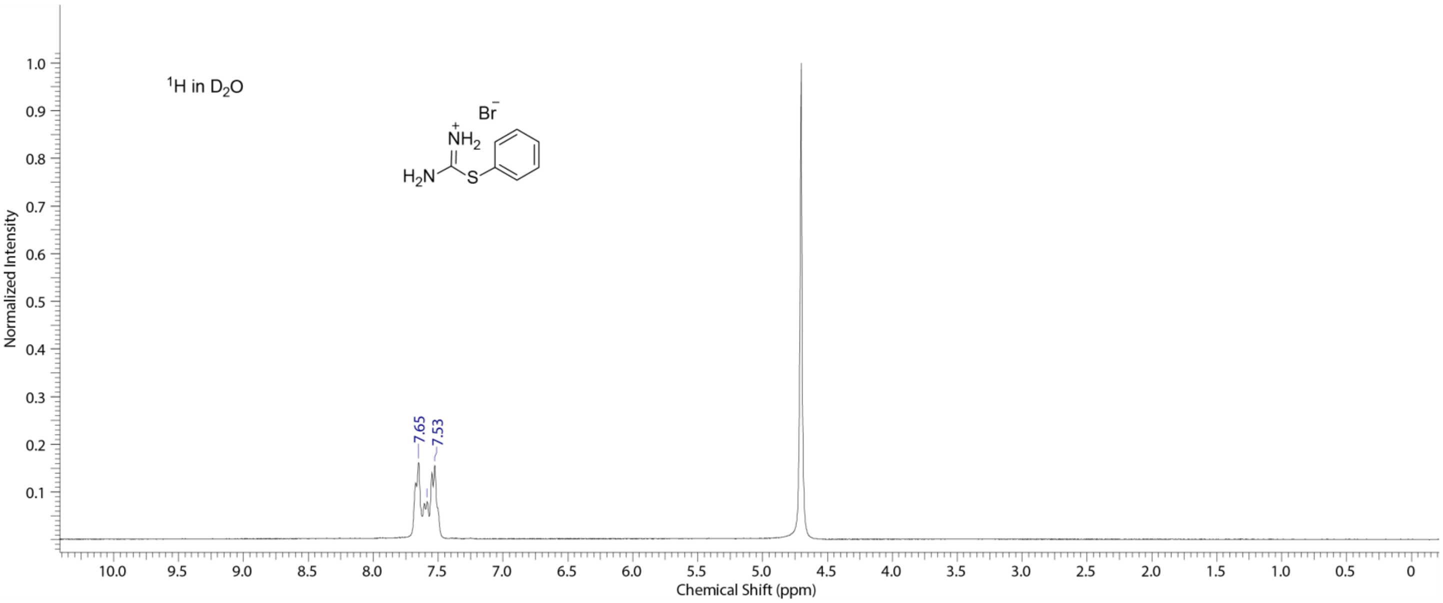

Supplementary Figure 45. ^{1}H NMR (300 MHz, D_2O) of the compound **4**.

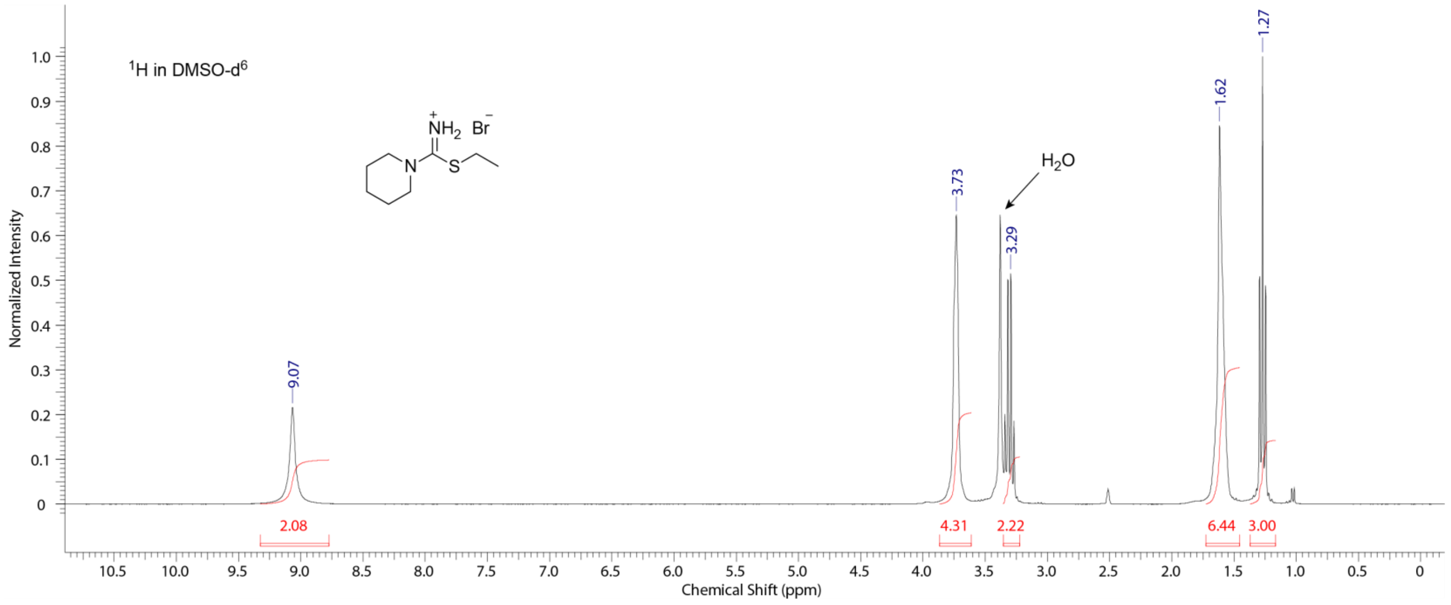

Supplementary Figure 46. ^{1}H NMR (300 MHz, DMSO-d^{6}) of the compound **7**.

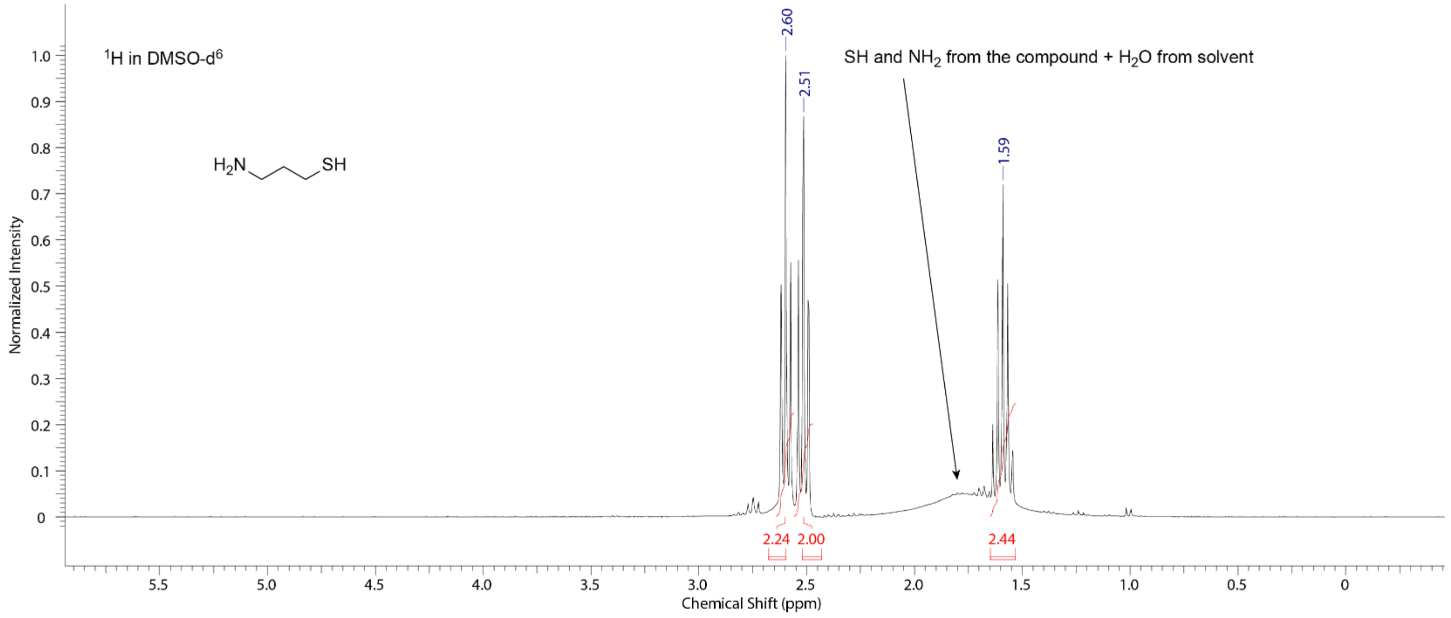

Supplementary Figure 47. ^{1}H NMR (300 MHz, DMSO-d^{6}) of the compound **9**.

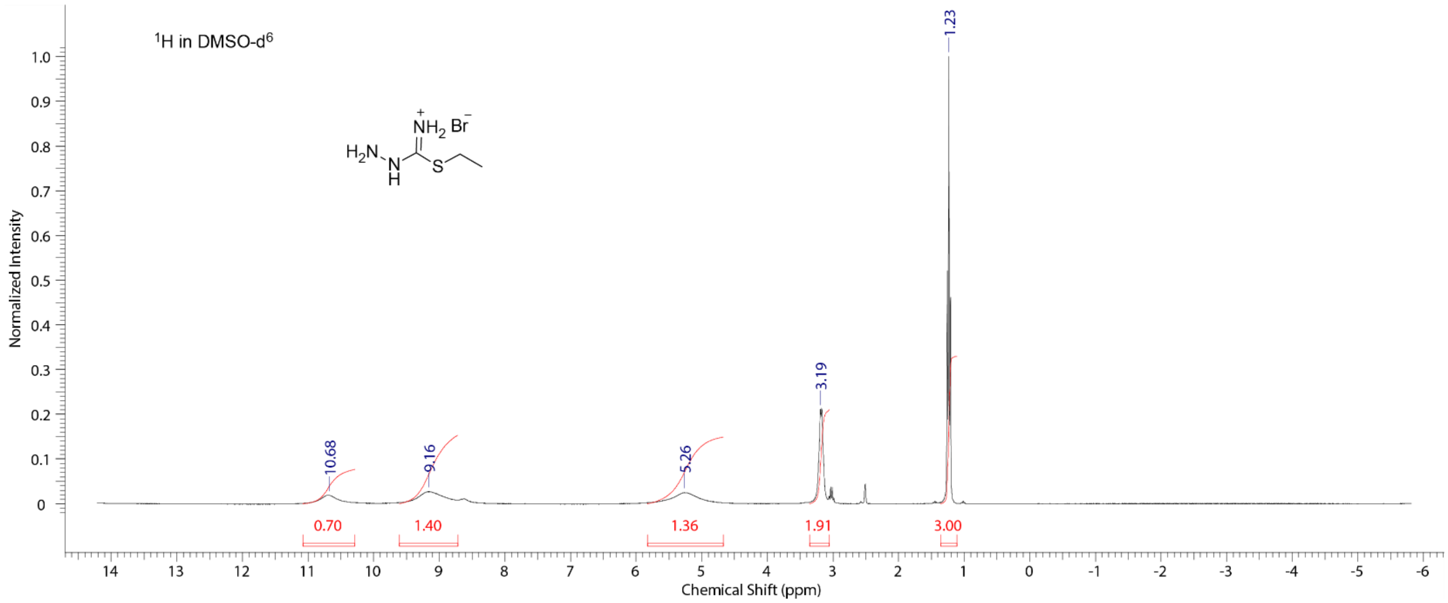

Supplementary Figure 48. ^{1}H NMR (300 MHz, DMSO-d^6) of the compound **6**.

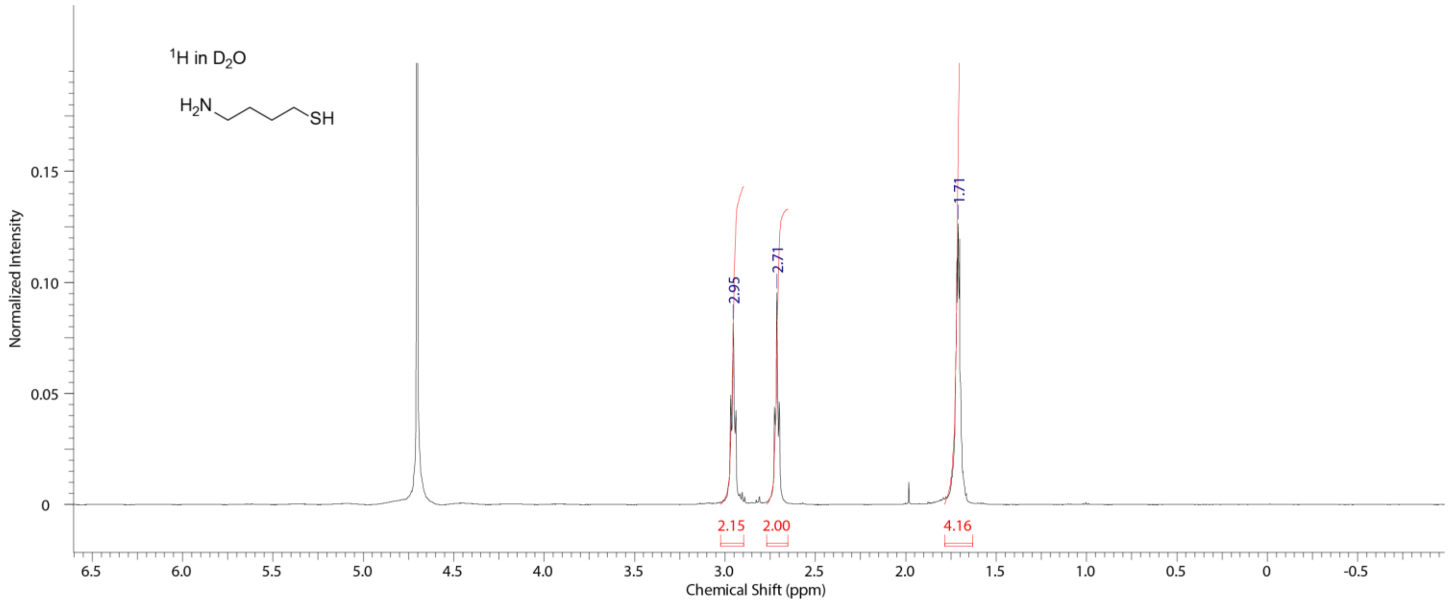

Supplementary Figure 49. ^{1}H NMR (300 MHz, D_2O) of the compound **10**.

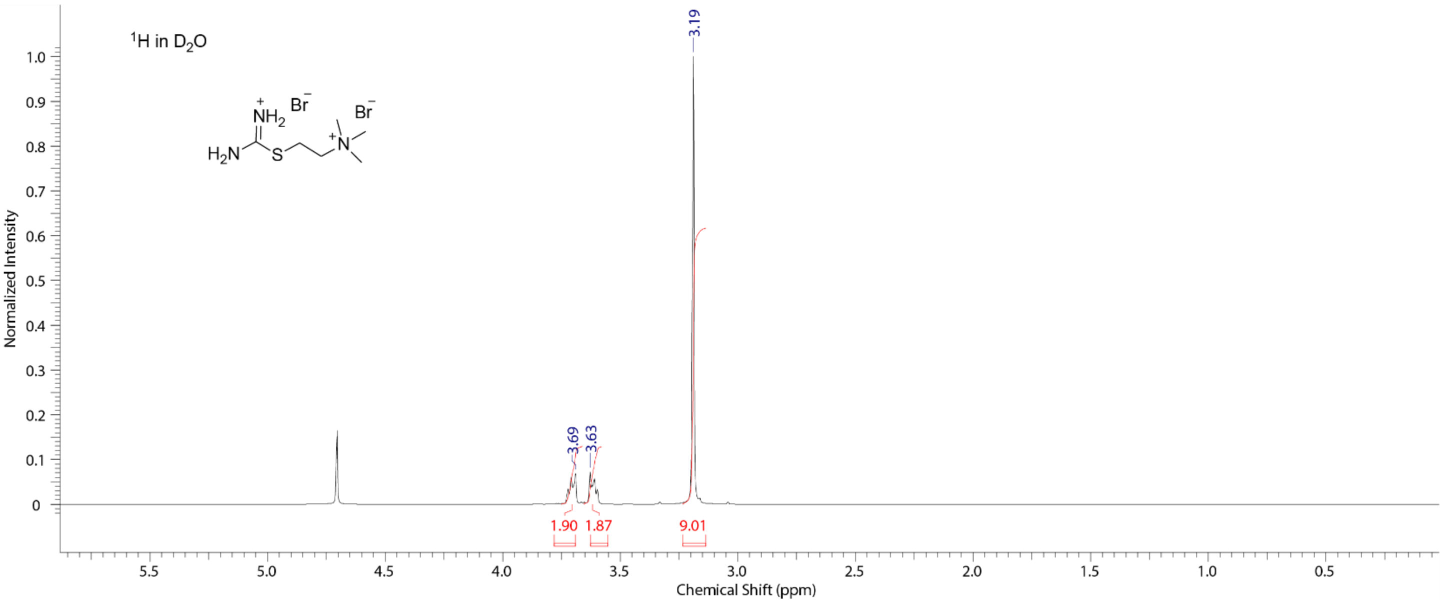

Supplementary Figure 50. ¹H NMR (500 MHz, D_2O) of the compound **8**.

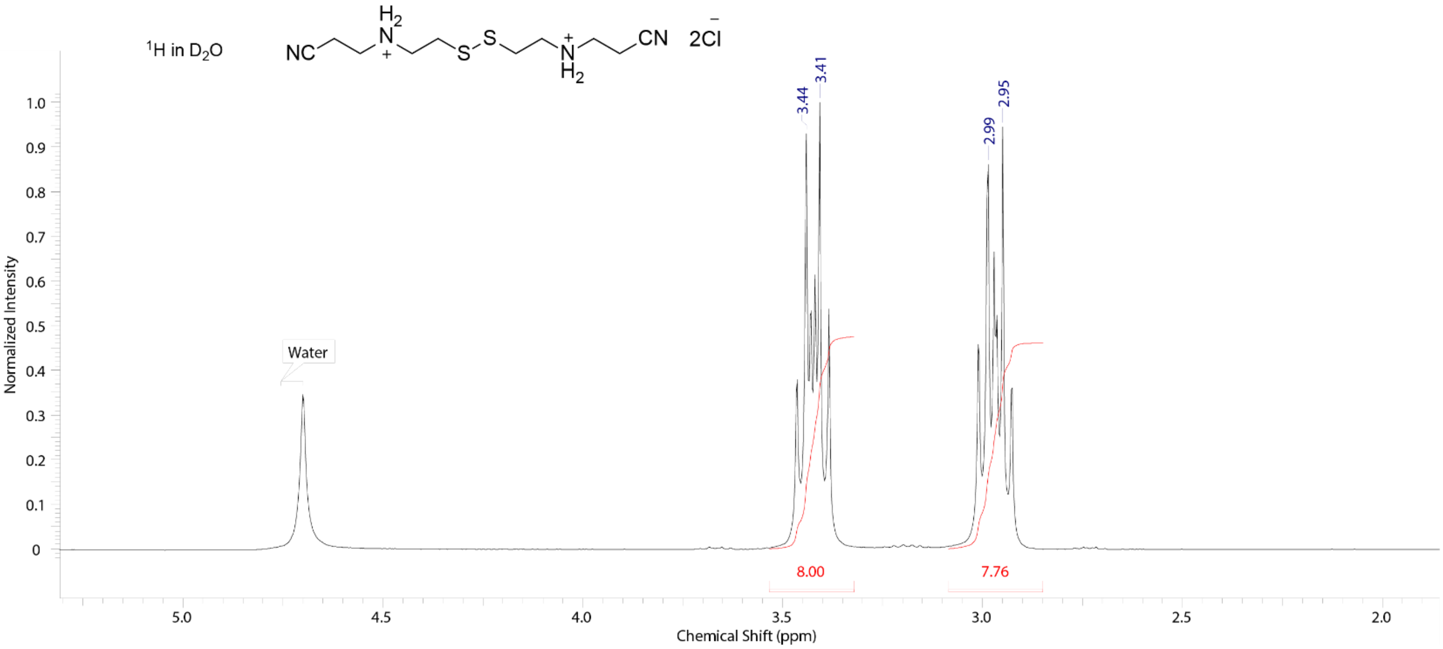

Supplementary Figure 50. ¹H NMR (300 MHz, D_2O) of the compound **18**.

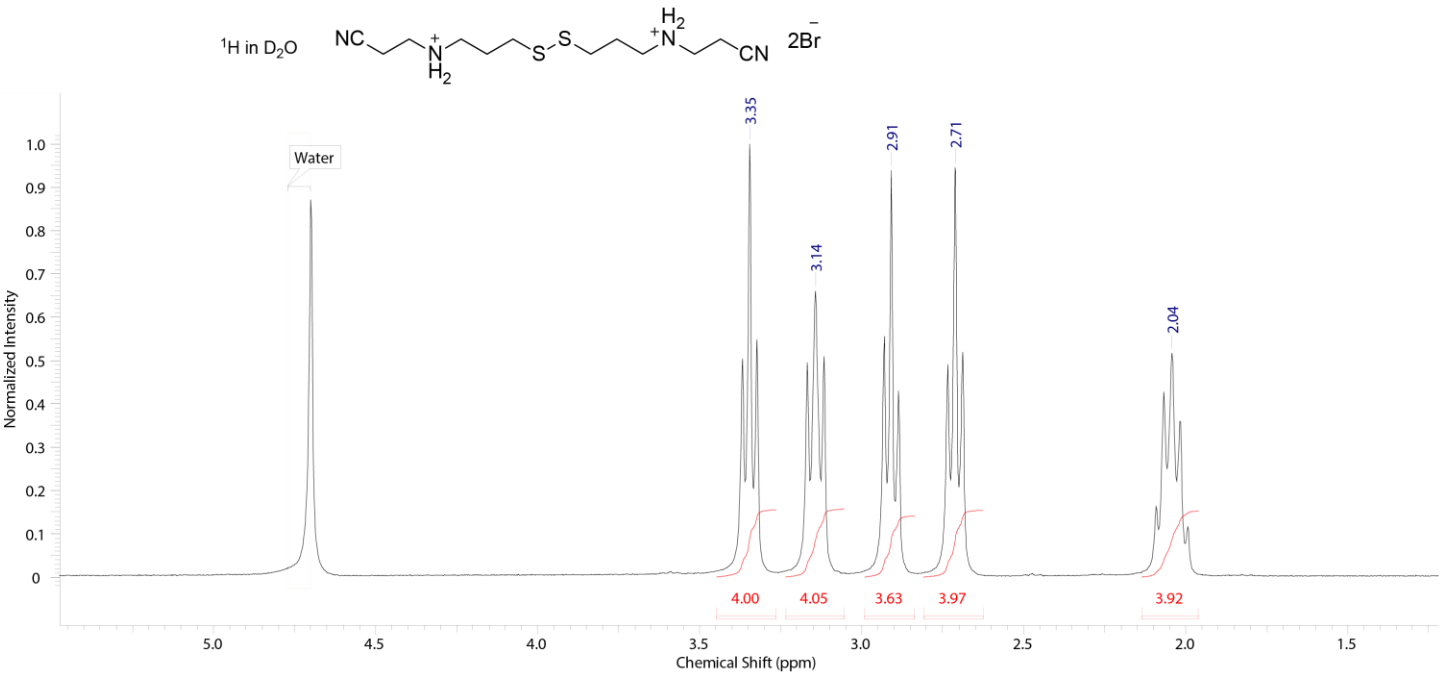

Supplementary Figure 50. ^{1}H NMR (300 MHz, D_2O) of the compound **19**.

9. Supplementary References

1. Brand, E. & Brand, F.C. Guanidoacetic Acid. *Organic Syntheses* **22** (1942).
2. Schramm, C.H., Lemaire, H. & Karlson, R.H. The Synthesis of Mercaptoalkanesulfonic Acids. *J. Am. Chem. Soc.* **77**, 6231-6233 (1955).
3. Pratt, R.F. & Bruice, T.C. Reactions of S-acylisothioureas. II. Effects of structure and stereochemistry on the rates of hydrolysis, thiol elimination, and S to N acyl migration in acylic systems. *J. Am. Chem. Soc.* **94**, 2823-2837 (1972).
4. Yokoyama, M., Ikuma, T., Obara, N. & Togo, H. Synthesis of mesoionic triazoline nucleosides. *J. Chem. Soc. Perk. T. 1* (1990).
5. Kong, X. et al. A mesogenic triphenylene-perylene-triphenylene triad. *Org. Lett.* **13**, 764-767 (2011).
6. Wieland, T. & Hornig, H. S-Acylspaltung beiS-Acetyl-ω-aminomercaptanen verschiedener Kettenlänge. *Justus Liebigs Annalen der Chemie* **600**, 12-22 (1956).
7. Yang, Y., Yin, B., Li, Q. & Englert, U. (2-Bromoethyl)trimethylammonium bromide and thiourea: A comparative study of three crystal structures. *J. Mol. Struct.* **920**, 401-408 (2009).
8. Raiziss, G.W. & Clemence, L.W. 2-Sulfanilyl-aminothiazoline. *J. Am. Chem. Soc.* **63**, 3124-3126 (1941).
9. Hoops, S. et al. COPASI- A COmplex PAthway SImulator. *Bioinformatics* **22**, 3067-3074 (2006).
10. Johnson, E.C. & Kent, S.B. Insights into the mechanism and catalysis of the native chemical ligation reaction. *J. Am. Chem. Soc.* **128**, 6640-6646 (2006).
11. Semenov, S.N. et al. Autocatalytic, bistable, oscillatory networks of biologically relevant organic reactions. *Nature* **537**, 656 - 660 (2016).
12. Semenov, S.N., Ainla, A., Skorb, E.V. & Postma, S.G.J. Four-Variable Model of an Enzymatic Oscillator Based on Trypsin. *Isr. J. Chem.* (2018).
